# Accelerating Subcutaneous Drug Development: A Mechanistic Absorption Model for the Open Systems Pharmacology Framework

**DOI:** 10.1002/psp4.70292

**Published:** 2026-06-25

**Authors:** Moriah Pellowe, Ilse Dubbelboer, Erik Sjögren

**Affiliations:** ^1^ Pharmetheus Uppsala Sweden; ^2^ Department of Pharmaceutical Biosciences Uppsala University Uppsala Sweden

**Keywords:** absorption, bioavailability, mechanistic modeling, MoBi, open systems pharmacology, pharmacokinetics, physiologically based biopharmaceutics modeling (PBBM), physiologically based pharmacokinetics (PBPK), PK‐Sim, subcutaneous drug delivery

## Abstract

This study describes the implementation of a mechanistic subcutaneous (SC) injection model for the Open Systems Pharmacology platform. As the SC route of administration is gaining increased popularity, there is a growing need for tools to predict, analyze, and understand the SC absorption process and the mechanisms involved. The interplay between molecular, formulation, administration, and physiological properties influences both the rate and extent of drug appearance in circulation. The primary objective of this study was to provide a structural modeling basis for mechanistic simulations of drug absorption after SC administration, considering fundamental molecular properties and systemic disposition characteristics. A key aspect of the model design was the intention to support generalizability and translational application across drug characteristics and species, providing a consistent structure for both small molecules and biologics. The SC model was implemented leveraging the structure and parameterization of PK‐Sim to allow unified integration to the whole‐body physiologically based pharmacokinetic model. An input‐response analysis and a set of case examples were conducted to visualize model responsiveness and illustrate potential application in drug development. The generic framework may also serve as the backbone for further implementations to describe complex injection and formulation dependencies. Collectively, this framework establishes a mechanistic foundation for the simulation of SC drug absorption of both small molecules and biologics, providing a basis for further development and informed evaluation across preclinical and clinical stages within the Open Systems Pharmacology platform.

## Study Highlights

- What is the current knowledge on the topic?
  ○ A challenge in the development of SC administered drugs is the translation of in vitro and preclinical in vivo data to clinical biopharmaceutics performance and pharmacokinetics. Establishing a unified mechanistic framework that captures the SC absorption profiles across diverse therapeutic classes remains an ongoing challenge in the field.
- What question did this study address?
  ○ The aim of this study was to provide a generic physiologically based SC absorption model applicable for both small molecules and biologics across species, incorporating mechanistic descriptions of local disposition and absorption processes.
- What does this study add to our knowledge?
  ○ This work establishes a mechanistic backbone within the Open Systems Pharmacology platform and illustrates the model's capability to capture the drug kinetics of a diverse set of drugs. The framework provides a structural basis for evaluating how drug properties and physiological factors influence SC drug absorption.
- How might this change drug discovery, development, and/or therapeutics?
  ○ By providing a structural basis to integrate physiological and biopharmaceutics factors influencing SC absorption, this model supports mechanistically informed simulations of pharmacokinetics, guidance of formulation strategies, and the optimization of dosing regimens.

## 1 Introduction

Subcutaneous (SC) administration is used for a variety of drug products, including small molecules, requiring immediate onset or formulated for extended release, and large biopharmaceuticals, like monoclonal antibodies, which cannot be administered via the gastrointestinal administration route [1]. Compared with intravenous administration, the SC route offers several advantages, including reduced invasiveness, greater convenience, and lower healthcare costs. However, it also presents certain limitations, such as restricted injection volume, potential injection‐site pain, and slower absorption [2, 3, 4]. For drugs with inherent limitations to intestinal absorption, for instance as a consequence of suboptimal oral biopharmaceutics properties or extensive pre‐systemic degradation, the SC route may provide an alternative means to achieve higher and more consistent levels of drug reaching the systemic circulation [5, 6]. In addition, the SC space has been proposed as a site for the administration of engineered drug delivery systems designed to meet specific therapeutic objectives, such as prolonged or controlled drug release [7, 8].

A remaining challenge for the development of SC administered drugs is to predict the clinical biopharmaceutics performance and pharmacokinetic behavior based on in vitro and preclinical in vivo studies [9]. To increase possibilities for translations and extrapolations, the development of mechanistic in silico models, for example, physiologically based pharmacokinetic (PBPK) modeling or biopharmaceutics modeling (PBBM), are undertaken [10, 11]. Integration of such models into the development process would offer significant advantages for both small and large molecule therapeutics administered via the SC route. By providing possibilities to mechanistically understand the physiological and biopharmaceutics factors influencing drug absorption and disposition, informed predictions of pharmacokinetics, guidance of formulation strategies, and support to the optimization of dosing regimens can be achieved.

Previously published mechanistic models aimed to describe the absorption from the SC site through the vascular or lymphatic pathway [10]. Models for small molecules generally described the SC administration site empirically, whereas models for biologics commonly contained a physiologically based SC administration site. In the biologics SC models, the one‐ or two‐pore theory was often applied, describing the mass transfer with both convection and diffusion. Despite these advances, challenges remain to establish a generalized framework to predict the biopharmaceutics and pharmacokinetic profiles across diverse therapeutic classes, highlighting the need for further efforts in this area [10].

Here we present an in silico framework to simulate and analyze the absorption and pharmacokinetic behavior of subcutaneously administered drugs. The specific aim was to provide the structural backbone of a generic physiologically based SC absorption model applicable for both small molecules and biologics across different species, including mechanistic descriptions of local disposition and absorption processes. The model was implemented in an open‐source PBPK platform and harmonized to this platform's standardized model structures and parameterization. This approach leverages established methodologies for systemic disposition and provides a consistent foundation for mechanistically‐informed evaluations across the drug development phases [12].

## 2 Methods

### 2.1 Data Collection

Pharmacokinetic data following SC administration of small and large molecules were collected from the literature. The following selection criteria were applied during data selection: (i) plasma‐concentration time profiles available in tables or in figures ready for digitalization, (ii) data from intravenous and SC administration of either a solution or a suspension formulation, (iii) data from different species available (preferably human, one rodent, and one non‐rodent preclinical species). A set of case examples of complex formulations was also included to expand the exploration of potential model application beyond the current mechanisms implemented. Physiological parameters in the model were generally informed from the generic database included in the PBPK platform PK‐Sim, specifically the fat tissue. Information from public sources was used to parameterize the implementation of the local and central lymphatic system, as this information was not available from the PK‐Sim database. Detailed information is summarized in Supporting Information S1.

### 2.2 Software

The SC model was implemented in the Open Systems Pharmacology software MoBi version 11.2 and R package ospsuite. The model equations were implemented using the MoBi interface, while the model execution and parameter identifications were performed using R version 4.2. PBPK models for the individual compounds were either downloaded from the Open Systems Pharmacology GitHub model repository or initially created in PK‐Sim version 9 and higher, making use of the default implementation for small or large molecules respectively and for respective species anatomical and physiological characteristics. These systemic PBPK models were then connected to the SC model to inform compound and physiological parameters. Pharmacokinetic reference data in graphical format were digitized using WebPlotDigitizer 4.8 (https://automeris.io/wpd/).

### 2.3 Subcutaneous Model Framework

**FIGURE 1:**
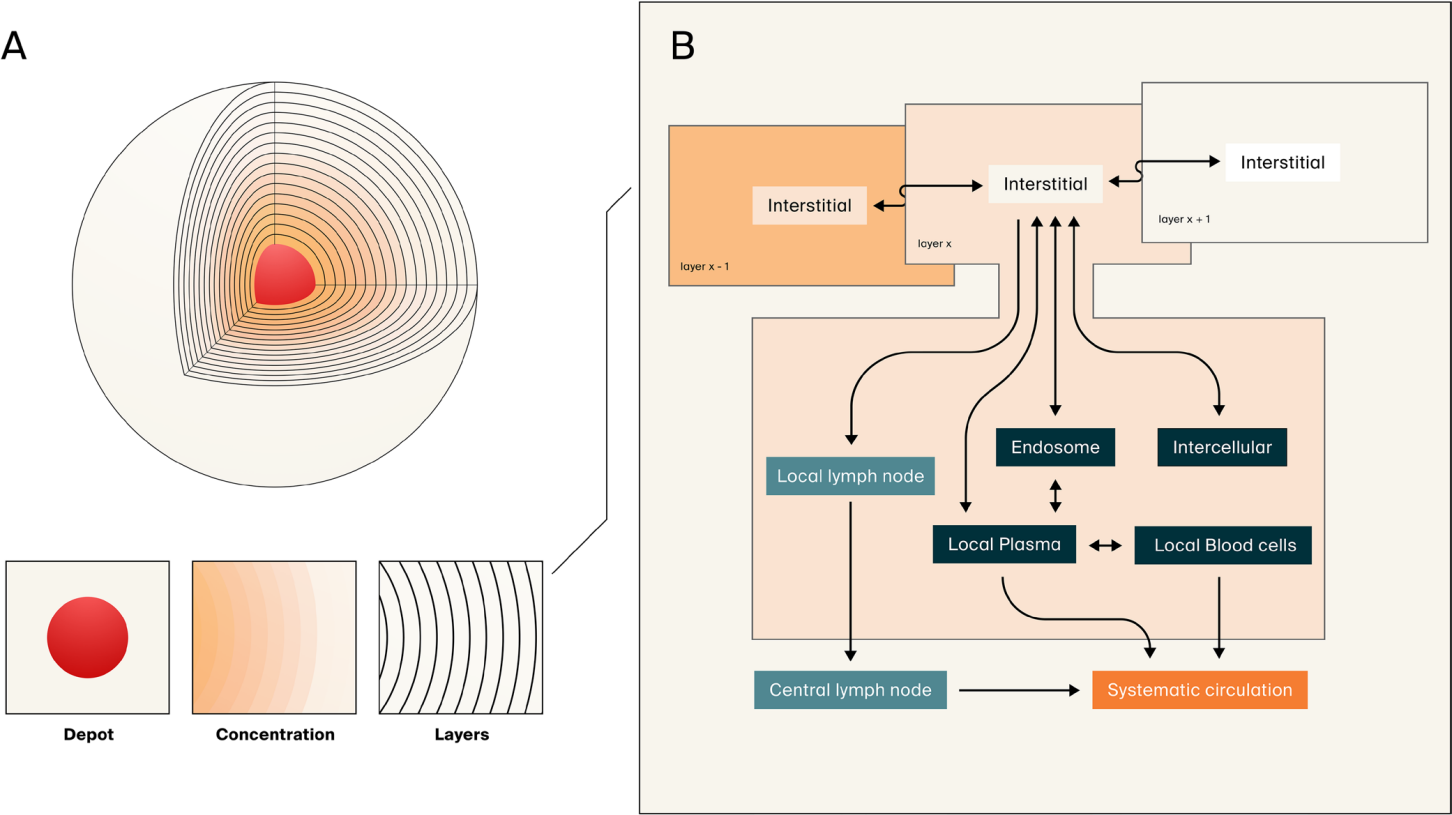
Graphical representation of the subcutaneous absorption and disposition model. (A) The physical injection site is represented by a volume including the injected formulation (depot in red) and the surrounding tissue organized in layers with a discrete thickness. At injection, part of the volume will form the depot while the rest, including solubilized drug, will disperse into the surrounding tissue. Spatiotemporal distribution of drug throughout the tissue is thereafter described by passive diffusion in the interstitial space. (B) Tissue organization, distribution, and description of mass transfer as implemented in PK‐Sim is adopted as a physiological representation for each tissue layer. Translocation both via cell membrane permeability and endothelial fenestration may occur dependent on the molecule's properties. Moreover, generic representation of endosomal functionalities (uptake, recirculation, clearance, and neonatal Fc receptor salvaging) as well as lymphatic drainage into local and subsequent central lymph nodes are represented within each layer's interstitial space. Systemic absorption rate and bioavailability are determined by the sum of said processes.

The physical injection site was represented in the model by a volume including the injected formulation and the surrounding tissue. A generic implementation to accommodate initial conditions as well as a mechanistic description of tissue disposition and uptake into vasculature and lymph was adopted to enable future customization of the model for specific cases. A graphical representation of the SC absorption and disposition model is depicted in Figure 1. The SC model builds on and is aligned with the default whole‐body PBPK implementation and parameterization in PK‐Sim. By directly linking selected compound‐specific and physiological parameters in the SC model to the systemic PBPK model, the SC model is automatically updated according to the specific species and drug considered. Further information on components, parameters assumptions, and default settings are summarized in Supporting Information S1. The SC model implementation is provided as a MoBi v12 module (Supporting Information S4).

The general model structure of the injection site was implemented as compartments with interconnecting flows involving a central depot, representing the injected formulation, and the surrounding SC tissue as discretized tissue layers to describe the spatiotemporal distribution of drug throughout the tissue. Initial drug administration deposits drug both as the central depot of either cylindrical or spherical geometry and as an immediate dispersion of solubilized drug within the SC tissue. The specified volume of the injection and level of dispersion informs geometric parameters of the depot and the tissue layers. The total injection site tissue depth was dynamically adjusted to ensure that the initial dispersion volume occupied no more than one‐third of the available tissue layers. Particle dissolution in the depot of solid particles was described according to the dissolution module in PK‐Sim. An implementation of precipitation of solubilized drug to solid particles was included to allow investigations of formulation stability and performance.

Following injection, solubilized drug in the depot will diffuse from the depot to the interstitial space of the innermost tissue layer while solid particles are retained within the depot volume. The surrounding layers of tissue are modeled in the same shape as the depot, either as concentric spheres or concentric cylinders, and serially interconnected via their interstitial spaces. Tissue organization, distribution, and description of mass transfer as implemented in PK‐Sim were adopted as a physiological representation for each tissue layer. This involved extravasation between the interstitial space and local vascularity via endothelial cell and fenestration permeability and via cell membrane permeability to the intracellular space dependent on the molecular properties of the drug. Representations of interstitial binding to plasma proteins, for example, albumin, as well as a placeholder for metabolic degradation in the interstitial space, were also included. Generic representation of endosomal functionalities, that is, uptake, recirculation, clearance, and neonatal Fc receptor (FcRn) mediated salvaging, as well as lymphatic drainage into local, and subsequent central lymph nodes, were represented within each layer's interstitial space. A local representation of the systemic framework for describing trafficking of FcRn and endogenous IgG was implemented to allow description of local processes, for example, injection site specific modulations and local saturation of FcRn. Systemic absorption rate and bioavailability were determined by the sum of the described lymphatic and vascular processes. After absorption, the systemic model governs the subsequent drug disposition process.

The model's initial conditions are governed by several key injection parameters, including dose volume, injection rate, drug solubility, depot geometry, and the extent of initial tissue dispersion. Many of these variables are inherently sensitive to formulation characteristics, specific injection conditions, and host physiological attributes. At present, generic implementations to automatically account for these dependencies are not yet integrated; consequently, the model requires explicit parameterization. This can be achieved through a priori assumptions, specialized in vitro or in situ experimental data, or via a “learn‐and‐confirm” strategy that leverages observed plasma concentration‐time profiles. While establishing generic rules or predictive correlation methods to these inputs remains a future objective, this was beyond the scope of the current study.

### 2.4 Subcutaneous Model Evaluation Strategy

The SC model was evaluated towards observed reference data from two monoclonal antibodies (mAbs), representing large molecules (adalimumab and rituximab) and five conventional small molecules (buprenorphine, fentanyl, lenacapavir, methotrexate, and ropivacaine). These molecules were selected based on the availability of pharmacokinetic data after both intravenous and SC administration. For each of the molecules, the intravenous data were used to develop the systemic disposition model. The SC model was then evaluated towards observed drug kinetics after SC administration. Different strategies were applied for large molecules as compared to small molecules and depending on the injected formulation.

To inform the SC model structure, all relevant parameters from the exported systemic disposition model, that is, pkml file exported from PK‐Sim, were transferred to the SC model template structure in MoBi. This operation was validated by comparing the output from the original PK‐Sim model to the results from the integrated PBPK‐SC model applying the same intravenous dose regimen. The informed SC model files were then used as the naïve starting points for simulations and evaluations towards observed pharmacokinetic data following SC administration of solutions or immediate release formulations.

Several aspects of information relevant to the absorption process were either not investigated or not disclosed in the included case studies. Consequently, the objective of this evaluation was to show general SC model performance and to illustrate potential context‐of‐use while specific validations would require more comprehensive datasets including detailed information on injection parameters, local interactions, and formulation characteristics. Identified parameters values should hence be viewed as qualitative indications of respective general processes and not as quantitative measures. For the large molecules (mAbs), the parameters injection dispersion factor, local endosomal uptake, local metabolism, and generic bioavailability (= dose delivered) were explored while for the conventional drug molecules, the local parameters for blood‐tissue partition, cellular permeability, and metabolism were addressed. To limit the parameter space of exploration for the conventional drug molecules, the general injection dispersion factor for each species was informed by the results of the large molecule exploration. Furthermore, for solution formulations, no precipitation in the injection site was allowed, assuming maintained solubilization of the drug after injection. Examples of formulation dependency investigations, for example, buprenorphine, and hypothesis driven explorations, for example, lenacapavir, were conducted considering processes on a case‐by‐case basis. The potential for a learn‐and‐confirm philosophy, that is, leveraging preclinical data for clinical predictions, was considered in all cases.

### 2.5 Systemic Disposition Models

A generic, species and drug agnostic, fit for purpose strategy was applied to establish systemic disposition PBPK models of the investigated drugs. Specific drug and species characteristics of the elimination processes were not considered, as the purpose of the systemic models was to inform physiological parameters of the SC model and to allow convolution of simulated SC absorption to plasma concentration‐time profiles. Fundamental drug properties, for example, molecular weight (MW) and fraction unbound in plasma, were collected from the public repository DrugBank online (https://go.drugbank.com/) (Supporting Information S2). The systemic distribution model was selected according to drug class, that is, “The standard model for small molecules” for the conventional drugs and “The model for proteins and large molecules” for included mAbs. Drug elimination was implemented as a generic liver clearance (CL_H_) process for small molecules and via the generic endosomal clearance and FcRn salvaging model for mAbs. Systemic disposition was optimized via estimation of distribution and elimination parameters by minimization of sum of squared residuals between simulated and reference plasma concentration using the inbuilt “Parameter Identification” (PI) functionality in PK‐Sim. For small molecules, the critical model parameters identified (and their boundaries used in PI) were lipophilicity (lowest‐highest reported value), glomerular filtration rate (0–1), plasma clearance (0–1000 mL/min), and unbound protein fraction (lowest‐highest reported value). This resulted in one compound with optimized parameters per species. The standard PK‐Sim calculation methods for tissue partition coefficients (Kp) and cellular permeability (Pc) were initially tested for all drugs. If an inadequate result was achieved, other distribution methods were evaluated. For large molecules, the critical model parameter identified was the equilibrium dissociation constant to the neonatal Fc receptor (Kd_FcRn_) in the endosomal space (0.01–100 μM). Final systemic model settings and parameters are summarized in Supporting Information S2.

### 2.6 Sensitivity Analysis

A set of local sensitivity simulations was undertaken to investigate how the incorporated processes govern the dynamic behavior of SC absorption as represented by the model. Simulations were performed for a theoretical conventional drug (MW = 300 g/mol, CL_H_ = 10 mL/min/kg, fraction unbound (fu) = 0.1, the logarithm of the partition coefficient (logP) = 2, Pc = 0.01 dm/min, SC Kp = 1, red blood cell partition coefficient (Kp.rbc) = 1) and a generic large molecule (MW = 150 kDa, fu = 1, logP = −5, Kd_FcRn_ = 1 μM, Pc = 0) at a SC dose of 1 μmol in 1 mL to a typical human individual (73 kg) assuming an initial tissue dispersion at injection of 50%. Model sensitivity to cell permeability (0.01–100 × 10^−4^ dm/min) and SC Kp (0.01–100) as well as the level of Kp.rbc (0.01–100) and fu (0.01–1) were investigated for the conventional small molecule. For the generic large molecule, model sensitivity was explored towards MW (5–500 kDa) and affinity to FcRn (0.01–100,000 μM). In addition, the route of absorption for the large molecule, that is, vascular and lymphatic, was further explored in respect of level of interstitial protein binding (0.01–1). Finally, the influence of injection volume (0.1–10 mL) and initial tissue dispersion at injection (0.1%–99.9%) were investigated for both the small and the large molecules.

## 3 Results

### 3.1 Sensitivity Analysis

**FIGURE 2:**
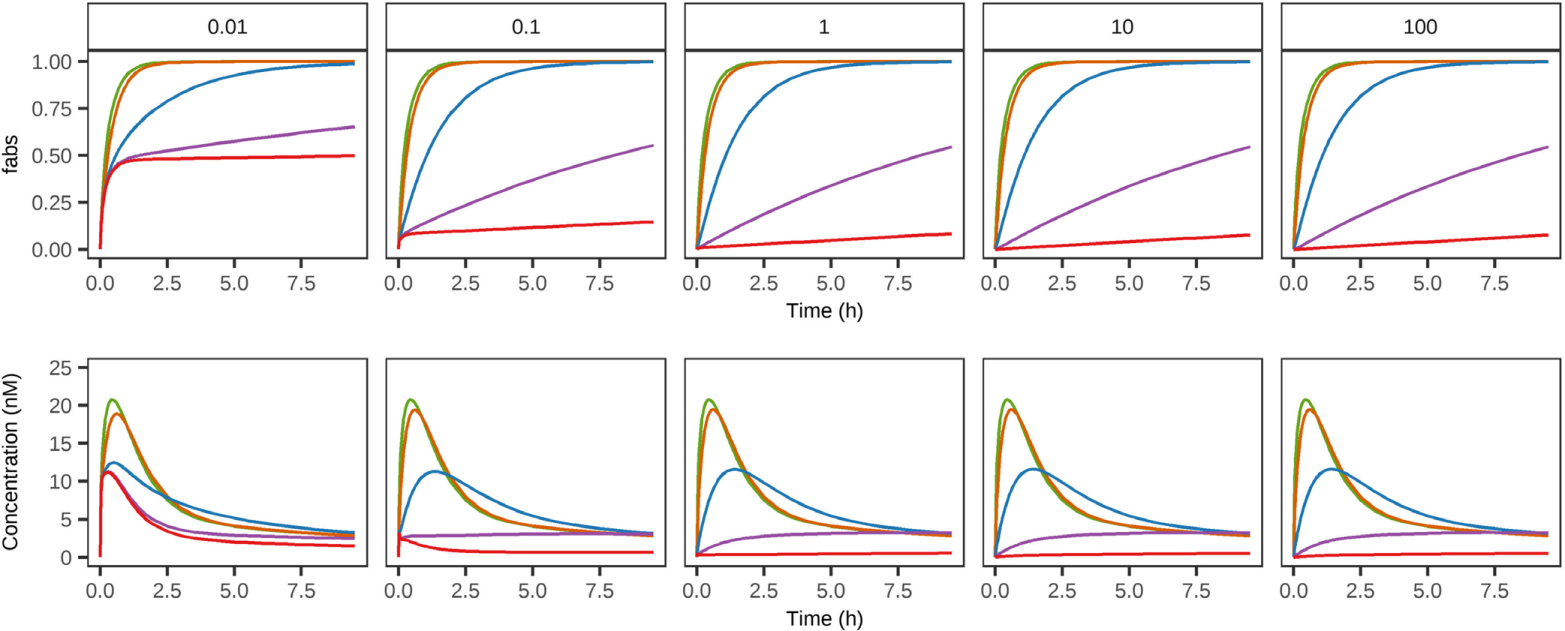
Model sensitivity to local tissue partition coefficient (Kp) and cellular permeability (Pc) for a generic small molecule. Simulated output as total fraction absorbed (fabs) and plasma concentration over time for a generic small molecule with variable cell permeability (0.01–100 × 10^−4^ dm/min, represented across columns) and Kp (green = 0.01, orange = 0.1, blue = 1, purple = 10, red = 100). For further information see Sensitivity Analysis in Methods.

**FIGURE 3:**
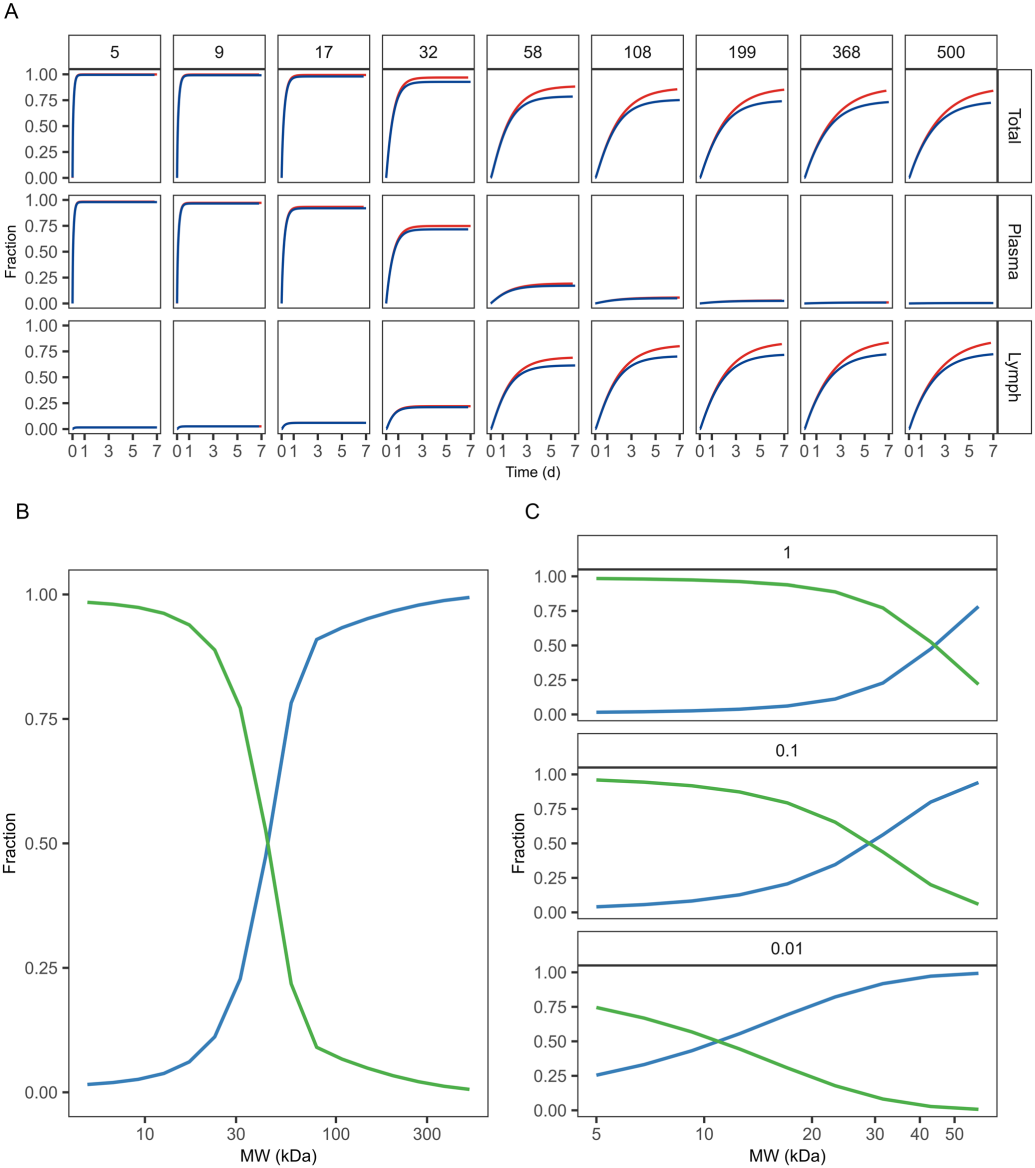
Model sensitivity to molecular weight for a generic large molecule. (A) Simulated fraction absorbed over time (Total), and separately for the vascular and lymphatic route, for large molecules of varying molecular weight (5–500 kDa, represented across columns) with a high (red, Kd_FcRn_ = 0.01 μM) and low (blue, Kd_FcRn_ = 10,000 μM) affinity to the neonatal Fc receptor. (B) The fraction of the simulated total absorption taking place either via plasma (green) or lymph (blue) as a function of molecular weight, where (C) illustrates the influence of plasma protein binding (fu = 1 (default), 0.1, and 0.01) on the route of absorption. For further information see Sensitivity Analysis in Methods.

For conventional small molecules, the results from the sensitivity analysis displayed the model's sensitivity to local tissue distribution (Kp and Pc) (Figure 2) as well as vascular distribution (fu and Kp.rbc) (Supporting Information S3). Drug retention in SC tissue was considerable already at Kp = 1, with substantial retention at Kp ≥ 10. However, tissue retention was governed by the balance between intracellular translocation and vascular clearance. Notably, reduced cellular permeability enhanced absorption efficiency, while simultaneously promoting intracellular sequestration. Moreover, in a nonlinear fashion, partitioning to red blood cells increased absorption rate while fraction unbound reduced it, further highlighting the nature of competitive rates in the model both in terms of local and vascular drug properties. The route of absorption from the injection site was highly dependent on molecular size: vascular absorption predominated at MW < 30 kDa, whereas lymphatic drainage became the dominant pathway at MW > 40 kDa. This size‐dependent pathway of absorption had a direct effect on the effective rate of systemic absorption (Figure 3). A similar dependence on MW was observed for pre‐systemic loss via endosomal degradation, which was further modulated by affinity for the neonatal Fc receptor (FcRn). Furthermore, an inverse correlation was observed between the extent of lymphatic absorption and the unbound fraction of the drug within the extracellular compartment (Figure 3C). Finally, both injection volume and tissue dispersion at the injection site significantly influenced the absorption rate for a generic large molecule while less so for a small molecule (Supporting Information S3).

### 3.2 Case Studies

#### 3.2.1 Large Molecules: Adalimumab and Rituximab

**FIGURE 4:**
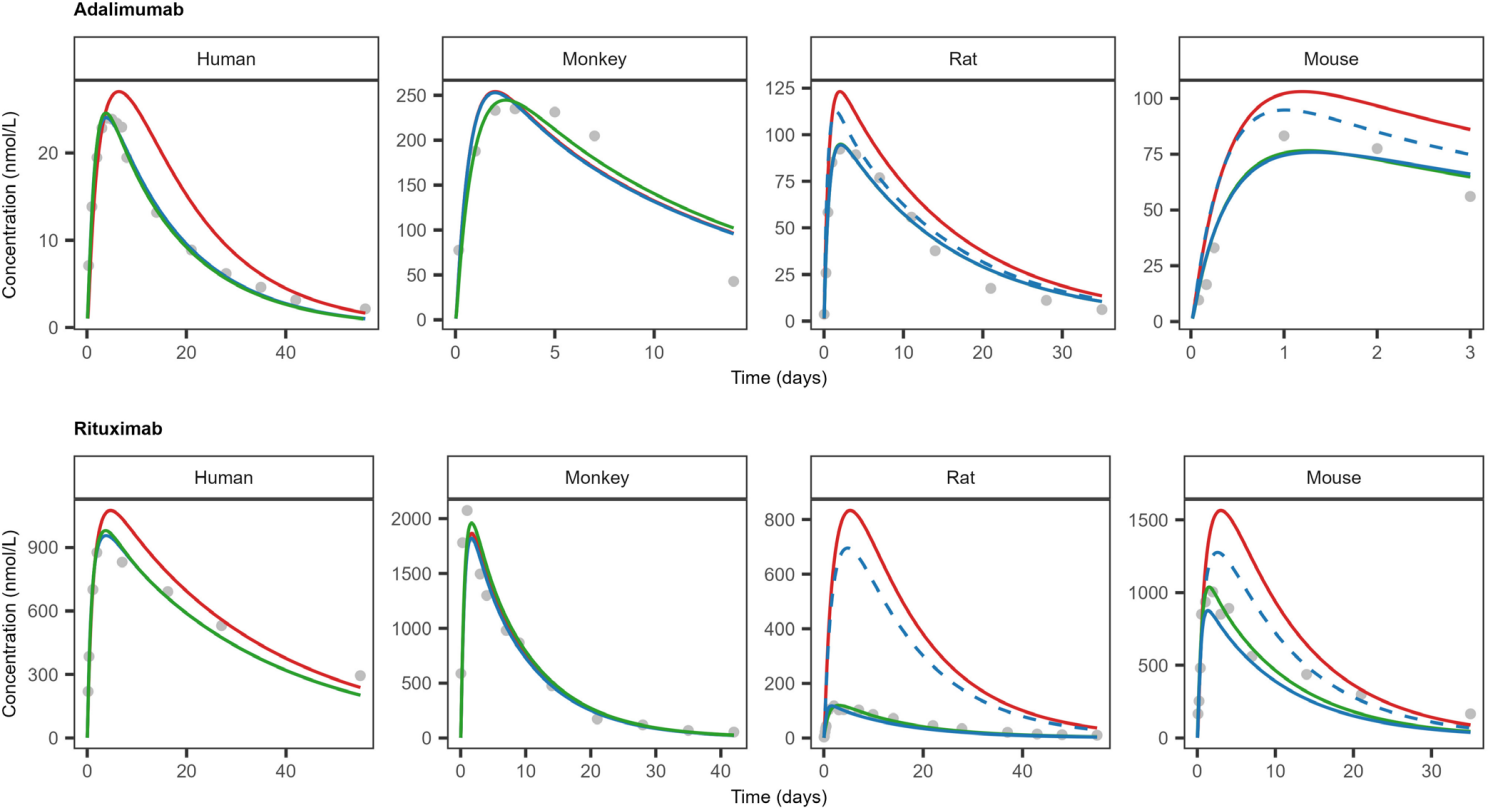
Observed (gray dots) and model simulated (solid lines) plasma concentration‐time profiles of adalimumab and rituximab in different species. For all species, model simulations were performed with three different optimization strategies: Dispersion only (red), dispersion and endosomal uptake (green), or dispersion and dose delivered (blue). Dashed lines indicate optimization performed with an upper boundary condition, that is, 5 × generic PK‐Sim value, to the endosomal uptake capacity. Observed reference data and study information were collated from studies indicated in Supporting Information S2. Parameter values, model settings and further details are summarized in Tables S2.2 and S2.3.

Acceptable model performance could be achieved by adjusting the tissue dispersion factor, local endosomal uptake, bioavailability, and local metabolism rate (Figure 4). The different configurations of parameters were optimized for each dataset showing species and drug specific similarities and differences as summarized in Table S2.3. For instance, adequate fits for humans and monkeys were achieved by optimizing dispersion and, where necessary, adjusting the endosomal uptake rate within five times its generic PK‐Sim value. Conversely, characterizing rodent observations required a greater degree of pre‐systemic loss than the five‐fold endosomal uptake adjustment could provide, a trend particularly evident for rituximab in rats. In humans, tissue dispersion was less pronounced for adalimumab compared to rituximab while the opposite trend was identified for rat and mouse. Similar high dispersion was observed for both mAbs in monkeys. Adjusting the local metabolism rate did not provide better means to capture observations and it was kept to 0 throughout the analysis.

#### 3.2.2 Informing Small Molecules From the Large Molecules

As the clinical formulation of rituximab, MabThera [13], includes recombinant human hyaluronidase, an enzyme used to increase the dispersion and absorption of coadministered substances when administered subcutaneously, the dispersion factor for adalimumab was used to inform the species‐specific dispersion factor per default. For species without this information, that is, rabbits and dogs, an average of estimates from the other species was applied. Also, since endosomal uptake is of negligible relevance for small molecules this parameter was kept at the default value in PK‐Sim. With the dispersion factor informed, different configurations of parameters were optimized for each dataset. The explored parameters included local tissue partition coefficients, cell permeability, and metabolism. Modification of the default tissue dispersion was also addressed for specific case examples, that is, sustained release of buprenorphine and injection volume dependence of lenacapavir.

#### 3.2.3 Small Molecules: Immediate Release

**FIGURE 5:**
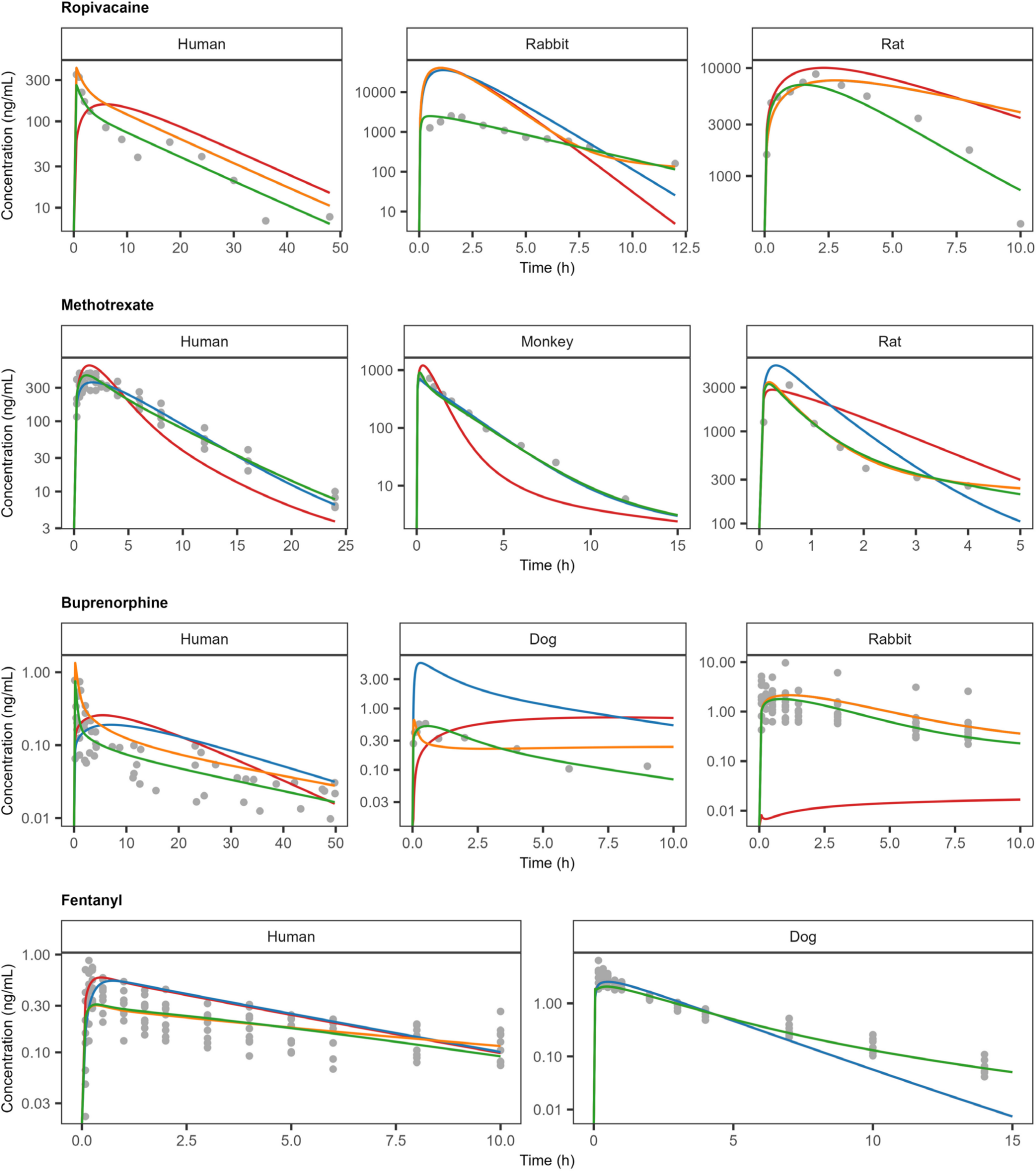
Observed (gray dots) and model simulated (solid lines) plasma concentration‐time profiles of ropivacaine, methotrexate, buprenorphine, and fentanyl after SC administration of formulations with expected immediate release. Model simulations were performed with naive parameterization (red) or different optimization strategies, tissue partitioning (blue), tissue partitioning and cell permeability (orange), or tissue partitioning, cell permeability, and local metabolism (green). Observed reference data and study information were collated from studies indicated in Supporting Information S2. Parameter values, model settings and further details are summarized in Tables S2.2 and S2.3.

Adequate description of observed data could be achieved by adjusting local parameters for Kp, Pc, and metabolic rate constant (kmet) (Figure 5). Drugs identified to be susceptible to SC metabolic degradation were consistent across species, with a few exceptions. However, due to the limited data set investigated, the results did not allow for additional establishment of general cross‐drug or cross‐species correlations or relationships. Still, for each specific drug, cross‐species trends towards the relevant processes could be identified as summarized in Table S2.3. For instance, fentanyl required a modulation in tissue distribution mediated by an increased Kp and a decreased Pc. Similarly, buprenorphine was successfully described across species by reducing the anticipated tissue distribution and introducing local presystemic degradation. Adequate performance was achieved for the hydrophilic drug methotrexate via a minor increase in tissue distribution. For ropivacaine, with local degradation included, the model was able to describe observations with only minor modulation to tissue distribution processes.

#### 3.2.4 Small Molecules: Formulation and Injection Effects

**FIGURE 6:**
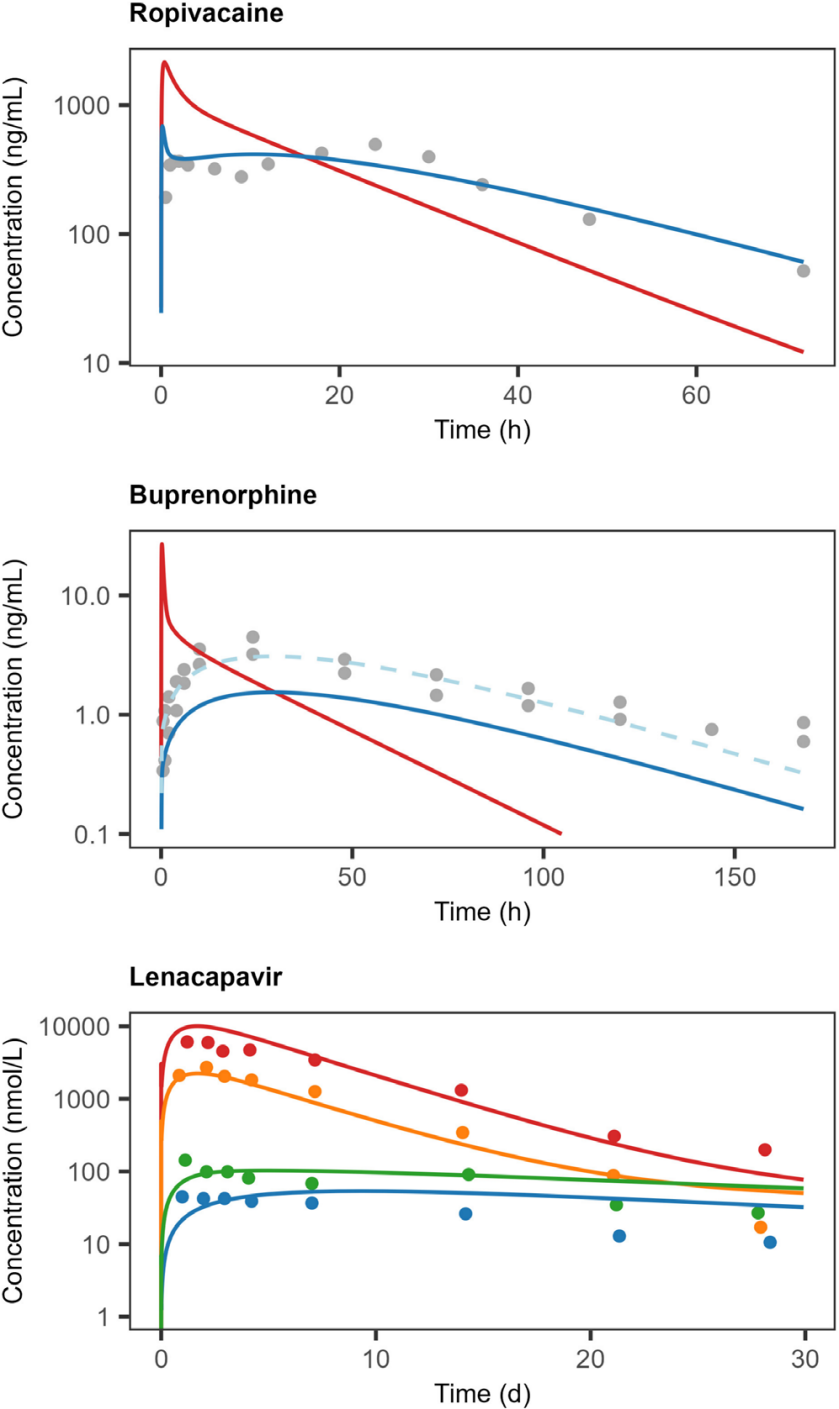
Observed (gray dots) and model simulated (solid lines) plasma concentration‐time profiles of ropivacaine, buprenorphine, and lenacapavir after SC administration of formulations with expected formulation‐controlled release. For ropivacaine and buprenorphine, model simulations were performed with parameterization informed from the immediate release investigations without (red) and with (blue) modification of release rate from the depot. To guide interpretations of buprenorphine release dynamics simulation, the simulation output × 2 was also displayed (light‐blue dashed line). Colors for lenacapavir indicated administration of 30 mg (blue), 60 mg (green), 300 mg (orange), and 1000 mg doses (red), simulated with different tissue dispersion at injection. Observed reference data and study information were collated from studies indicated in Supporting Information S2. Parameter values, model settings and further details are summarized in Tables S2.2 and S2.3.

The optimal configuration for tissue distribution and local metabolism identified in the immediate release investigation was carried over to the formulation and injection effects evaluation for ropivacaine and buprenorphine. For lenacapavir, an exploratory approach was adopted with the aim to apply the model for hypothesis generation. Simulation results were performed by modifying formulation and injection parameters while retaining molecular characteristics (Figure 6).

The formulation investigated for ropivacaine was a multivesicular liposome (MVL) preparation with reported human data [14]. The preparation was assumed to disperse as a solution at the injection where the fraction of formulation retained in the depot would maintain its integrity while the dispersed fraction would immediately be released. Furthermore, the drug's diffusion coefficient in the depot was used as a surrogate for formulation release rate, assuming that the rate of release would be slower than the drug's inherent diffusion rate. A similar approach was adopted for buprenorphine, for which clinical data on a formulation undergoing in situ transformation into a liquid crystalline gel phase was used as a case example [15]. In similarity to the MVL investigation, it was assumed that the drug's diffusion in the depot could be used as a surrogate for formulation release, however given the properties of this preparation, it was, in contrary to the MVL, assumed that no tissue dispersion occurred at injection. In the case of lenacapavir, hypothetical scenarios were generated to explain dose volume dependent drug kinetics after SC administration of an aqueous suspension formulation in dogs [16]. Observed pharmacokinetic differences between doses, that is, dose volumes, were captured by assuming increased tissue dispersion with the increased injection volumes administered (0.3, 0.6, 3, and 10 mL). In absence of formulation information, the presented results were achieved by applying a slower diffusion coefficient in the depot as a surrogate for particle dissolution and by setting the tissue dispersion to 5%, 10%, 66%, and 90%, as a consequence of the increasing injection volume.

## 4 Discussion

The primary aim of this work was to provide the backbone of a generic physiologically based SC absorption model applicable for both small molecules and biologics across different species, including mechanistic descriptions of local disposition and absorption processes. Implementing this model within the Open Systems Pharmacology (OSP) platform leverages established methodologies for systemic disposition to inform the SC model, facilitating its use across drug development phases. The results presented herein demonstrate the capacity of this mechanistic framework to capture the absorption kinetics of a diverse set of compounds, ranging from small molecules like fentanyl and methotrexate to large monoclonal antibodies such as adalimumab and rituximab. This mechanistic approach seeks to broaden the scope of SC absorption modeling within the existing whole‐body PBPK framework by providing the means to account for the nuances of the subcutaneous environment. Specifically, it enables the integration of injection‐site variables and local tissue kinetics, offering a more granular perspective on the drivers of systemic absorption and bioavailability.

Historically, modeling approaches for SC administration have often been bifurcated based on molecular size. Small molecule absorption has frequently been described using empirical models (e.g., first‐order absorption) which, while computationally efficient, lack mechanistic insight into local tissue disposition [10]. Conversely, models for biologics have typically incorporated more physiological details, often relying on the one‐ or two‐pore theory to describe mass transfer via convection and diffusion [4, 9]. However, these models have rarely been seamlessly integrated into a holistic PBPK framework adaptable to both drug classes.

The OSP implementation presented here distinguishes itself by harmonizing the SC site with the whole‐body PBPK structure in PK‐Sim [17, 18]. Unlike standalone mechanistic models such as SubQ‐Sim [11], which focus heavily on injection system parameters, our framework utilizes the native structure and parametrization of PK‐Sim to drive local disposition. This integration allows for a direct and harmonized translation of drug properties, such as lipophilicity and MW, between systemic disposition and the injection site, thereby enhancing computational efficiency and adaptability. This generic applicability addresses a critical gap identified in recent reviews [10], providing a structural middle ground that balances the rigorous mechanistic detail required for formulation analysis with the physiological flexibility needed for cross‐species translation. Moreover, once the SC absorption model has been established, this link facilitates direct subsequent analyses by leveraging the pallet of applications provided in PK‐Sim, such as drug–drug interactions and renal impairment assessments.

Through the included case examples, we illustrate how the model responds to different drug and system inputs. These simulations captured the absorption kinetics of a variety of large and small molecules with modulation of only a few parameters not already informed by the structural implementation. While these examples do not constitute a formal validation, they demonstrate the framework's ability to replicate observed absorption kinetics and resulting plasma concentration profiles. Key findings indicated that for large molecules, considerable interspecies differences, but also similarities, in dispersion and presystemic loss may exist, whereas for small molecules, cross‐species trends in local tissue distribution were observed, although the available data did not allow for delineation of discrete parameters. Importantly, the model correctly predicted the shift in absorption route from vascular to lymphatic dominance based on molecular weight, consistent with established physiological principles [19, 20]. Furthermore, it was shown that formulation investigations could be informed by carrying over information on local distribution previously identified from solution administrations and that injection volume dependencies could be explained by level of dispersion. However, it should be noted that the results were achieved with a surrogate approach for formulation drug distribution and release from the depot.

While the model was able to describe absorption dynamics and the plasma concentration‐time profiles for the investigated case studies, several limitations must be acknowledged. A primary challenge encountered during model evaluation was the lack of complete and detailed data in the public domain regarding injection parameters, for example, initial tissue dispersion, and formulation properties, for example, maintained solubilization. As noted in the methods, this necessitated the use of surrogate parameters or assumptions, such as retained solubilization, species‐specific harmonization in dispersion, and approximating formulation release via diffusion coefficients, which limited the granularity of mechanistic conclusions in specific cases. Furthermore, the sensitivity analysis highlighted that for small molecules, the model was highly sensitive to local Kp and Pc. While this confirmed the mechanistic relevance of these parameters, it also underscored a potential identifiability issue when simultaneous experimental data on local tissue concentrations are missing. Further investigation is required to determine how local parameters can be most accurately informed, whether through drug‐specific physicochemical properties, systemic kinetic data, or experimental measurements. Physiological assumptions, such as the homogeneity of the discretized tissue layers and the generic representation of endosomal functionalities, may also oversimplify the complex heterogeneity of the SC space [2]. Consequently, while the investigations conducted within the scope of this study provide a “proof‐of‐concept” for the model backbone, specific validations to each context of use are necessary. This is particularly critical for the mechanistic assessment of prolonged‐release formulations, as each delivery strategy requires a unique characterization of its release behavior and physiological interactions. However, given that this framework is an open‐source resource, researchers can extend its functionality with custom implementations and integrate proprietary data as required.

Future developments should focus on several key mechanistic extensions to enhance predictive accuracy. Specifically, incorporating explicit descriptions of drug‐excipient interactions, drug aggregation, and precipitation events within the depot is crucial. While a basic precipitation module was implemented in the current framework, further refinement is needed to mechanistically link in vitro precipitation kinetics to in vivo performance. Additionally, detailed modeling of drug interactions with the extracellular matrix is warranted, particularly regarding charge‐based interactions with hyaluronic acid or collagen, which can significantly retard absorption for certain biologics [9]. Beyond molecular interactions, the integration of time‐dependent physiological responses to the injection, such as inflammation, rubor, and local changes in blood flow and lymphatic drainage rates, would improve upon current static parameterizations. The framework also invites exploration into special population adaptations, addressing physiological alterations in blood flow or fat content found in pediatrics, obese patients, or specific disease states. Ultimately, this framework possesses the potential for integration into clinical decision‐support tools, enabling individualized therapy planning by evaluating the impact of injection volume, site, and formulation on therapeutic outcomes.

## 5 Conclusion

This work presents a structural foundation for physiologically based SC absorption modeling within the OSP suite. By illustrating that the framework can capture the absorption kinetics of a variety of large and small molecules, we have established a generic mechanistic backbone for this route of administration. While further evaluations to validate the model's performance for specific applications remain necessary, the proposed model provides a mechanistic framework to analyze the physiological and biopharmaceutics factors influencing SC absorption. It serves as a transparent starting point for physiologically based and mechanistic simulations of SC pharmacokinetics, guiding formulation strategies and optimizing dosing regimens for this administration route.

## Author Contributions

M.P., I.D., and E.S. wrote the manuscript. E.S. designed the research. M.P., I.D., and E.S. performed the research. M.P., I.D., and E.S. analyzed the data.

## Funding

This work was supported by Pharmetheus and The Swedish Drug Delivery Center (SweDeliver), a competence center funded by Vinnova (Sweden's Innovation Agency, Dnr 2024‐03851).

## Conflicts of Interest

All authors use Open Systems Pharmacology software, tools, or models in their professional roles.

## Supporting information


**Data S1:** psp470292‐sup‐0001‐DataS1.zip. **Supporting Information S1:** Subcutaneous Model.
**Supporting Information S2:** Simulation Information and Reference Studies
**Supporting Information S3:** Sensitivity Analyses.

## References

[1] I. R. Dubbelboer and E. Sjögren , “Overview of Authorized Drug Products for Subcutaneous Administration: Pharmaceutical, Therapeutic, and Physicochemical Properties,” European Journal of Pharmaceutical Sciences 173 (2022): 106181. 35381330 10.1016/j.ejps.2022.106181

[2] B. Bittner , W. Richter , and J. Schmidt , “Subcutaneous Administration of Biotherapeutics: An Overview of Current Challenges and Opportunities,” BioDrugs 32 (2018): 425–440. 30043229 10.1007/s40259-018-0295-0 PMC6182494

[3] R. Mathaes , A. Koulov , S. Joerg , and H. C. Mahler , “Subcutaneous Injection Volume of Biopharmaceuticals‐Pushing the Boundaries,” Journal of Pharmaceutical Sciences 105 (2016): 2255–2259. 27378678 10.1016/j.xphs.2016.05.029

[4] M. R. Turner and S. V. Balu‐Iyer , “Challenges and Opportunities for the Subcutaneous Delivery of Therapeutic Proteins,” Journal of Pharmaceutical Sciences 107 (2018): 1247–1260. 29336981 10.1016/j.xphs.2018.01.007 PMC5915922

[5] S. Mitragotri , P. A. Burke , and R. Langer , “Overcoming the Challenges in Administering Biopharmaceuticals: Formulation and Delivery Strategies,” Nature Reviews. Drug Discovery 13 (2014): 655–672. 25103255 10.1038/nrd4363 PMC4455970

[6] M. Sugihara , S. Takeuchi , M. Sugita , K. Higaki , M. Kataoka , and S. Yamashita , “Analysis of Intra‐ and Intersubject Variability in Oral Drug Absorption in Human Bioequivalence Studies of 113 Generic Products,” Molecular Pharmaceutics 12 (2015): 4405–4413. 26568266 10.1021/acs.molpharmaceut.5b00602

[7] H. Lou , M. Feng , and M. J. Hageman , “Advanced Formulations/Drug Delivery Systems for Subcutaneous Delivery of Protein‐Based Biotherapeutics,” Journal of Pharmaceutical Sciences 111 (2022): 2968–2982. 36058255 10.1016/j.xphs.2022.08.036

[8] A. Bauer , P. Berben , S. S. Chakravarthi , et al., “Current State and Opportunities With Long‐Acting Injectables: Industry Perspectives From the Innovation and Quality Consortium ‘Long‐Acting Injectables’ Working Group,” Pharmaceutical Research 40 (2023): 1601–1631. 36811809 10.1007/s11095-022-03391-y

[9] M. Sánchez‐Félix , M. Burke , H. H. Chen , C. Patterson , and S. Mittal , “Predicting Bioavailability of Monoclonal Antibodies After Subcutaneous Administration: Open Innovation Challenge,” Advanced Drug Delivery Reviews 167 (2020): 66–77. 32473188 10.1016/j.addr.2020.05.009

[10] I. R. Dubbelboer and E. Sjögren , “Physiological Based Pharmacokinetic and Biopharmaceutics Modelling of Subcutaneously Administered Compounds—An Overview of In Silico Models,” International Journal of Pharmaceutics 621 (2022): 121808. 35533921 10.1016/j.ijpharm.2022.121808

[11] X. J. H. Pepin , I. Grant , and J. M. Wood , “SubQ‐Sim: A Subcutaneous Physiologically Based Biopharmaceutics Model. Part 1: The Injection and System Parameters,” Pharmaceutical Research 40 (2023): 2195–2214. 37634241 10.1007/s11095-023-03567-0 PMC10547635

[12] J. Lippert , R. Burghaus , A. Edginton , et al., “Open Systems Pharmacology Community—An Open Access, Open Source, Open Science Approach to Modeling and Simulation in Pharmaceutical Sciences,” CPT: Pharmacometrics & Systems Pharmacology 8 (2019): 878–882. 31671256 10.1002/psp4.12473 PMC6930856

[13] Roche Pharma AG , “MabThera: EPAR—Product Information,” (2009), https://www.ema.europa.eu/en/documents/product‐information/mabthera‐epar‐product‐information_en.pdf.

[14] Y. Shen , Y. Ji , S. Xu , D. Q. Chen , and J. Tu , “Multivesicular Liposome Formulations for the Sustained Delivery of Ropivacaine Hydrochloride: Preparation, Characterization, and Pharmacokinetics,” Drug Delivery 18 (2011): 361–366. 21428705 10.3109/10717544.2011.557788

[15] M. Albayaty , M. Linden , H. Olsson , M. Johnsson , K. Strandgården , and F. Tiberg , “Pharmacokinetic Evaluation of Once‐Weekly and Once‐Monthly Buprenorphine Subcutaneous Injection Depots (CAM2038) Versus Intravenous and Sublingual Buprenorphine in Healthy Volunteers Under Naltrexone Blockade: An Open‐Label Phase 1 Study,” Advances in Therapy 34 (2017): 560–575. 28070862 10.1007/s12325-016-0472-9

[16] R. Subramanian , J. Tang , J. Zheng , et al., “Lenacapavir: A Novel, Potent, and Selective First‐in‐Class Inhibitor of HIV‐1 Capsid Function Exhibits Optimal Pharmacokinetic Properties for a Long‐Acting Injectable Antiretroviral Agent,” Molecular Pharmaceutics 20 (2023): 6213–6225. 37917742 10.1021/acs.molpharmaceut.3c00626 PMC10698746

[17] S. Willmann , J. Lippert , M. Sevestre , J. Solodenko , F. Fois , and W. Schmitt , “PK‐Sim: A Physiologically Based Pharmacokinetic ‘Whole‐Body’ Model,” Biosilico 1 (2003): 121–124.

[18] C. Niederalt , L. Kuepfer , J. Solodenko , et al., “A Generic Whole Body Physiologically Based Pharmacokinetic Model for Therapeutic Proteins in PK‐Sim,” Journal of Pharmacokinetics and Pharmacodynamics 45 (2018): 235–257. 29234936 10.1007/s10928-017-9559-4 PMC5845054

[19] A. Supersaxo , W. R. Hein , and H. Steffen , “Effect of Molecular Weight on the Lymphatic Absorption of Water‐Soluble Compounds Following Subcutaneous Administration,” Pharmaceutical Research 7 (1990): 167–169. 2137911 10.1023/a:1015880819328

[20] C. J. H. Porter and S. A. Charman , “Lymphatic Transport of Proteins After Subcutaneous Administration,” Journal of Pharmaceutical Sciences 89 (2000): 297–310. 10707011 10.1002/(SICI)1520-6017(200003)89:3<297::AID-JPS2>3.0.CO;2-P

