# Supplementary material for: Accelerating Subcutaneous Drug Development: A Mechanistic Absorption Model for the Open Systems Pharmacology Framework
Source: CPT Pharmacometrics Syst Pharmacol. 2026 Jun 25;15(7):e70292. doi: 10.1002/psp4.70292 (PMC13296821; doi:10.1002/psp4.70292)
Supplement: Supplementary file 1 — Data S1: psp470292‐sup‐0001‐DataS1.zip. Supporting Information S1: Subcutaneous Model. Supporting Information S2: Simulation Information and Reference Studies Supporting Information S3: Sensitivity Analyses. [file PSP4-15-e70292-s001.zip › PSP-2026-0008-s01.docx]

Supporting Information S1: Subcutaneous Model
Accelerating Subcutaneous Drug Development: A Mechanistic Absorption Model for the Open Systems Pharmacology Framework

1. Model diagram

| a  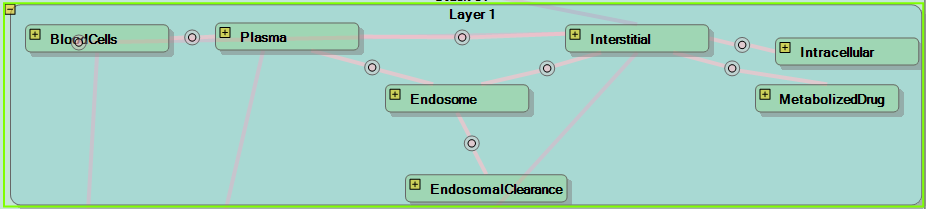 | c 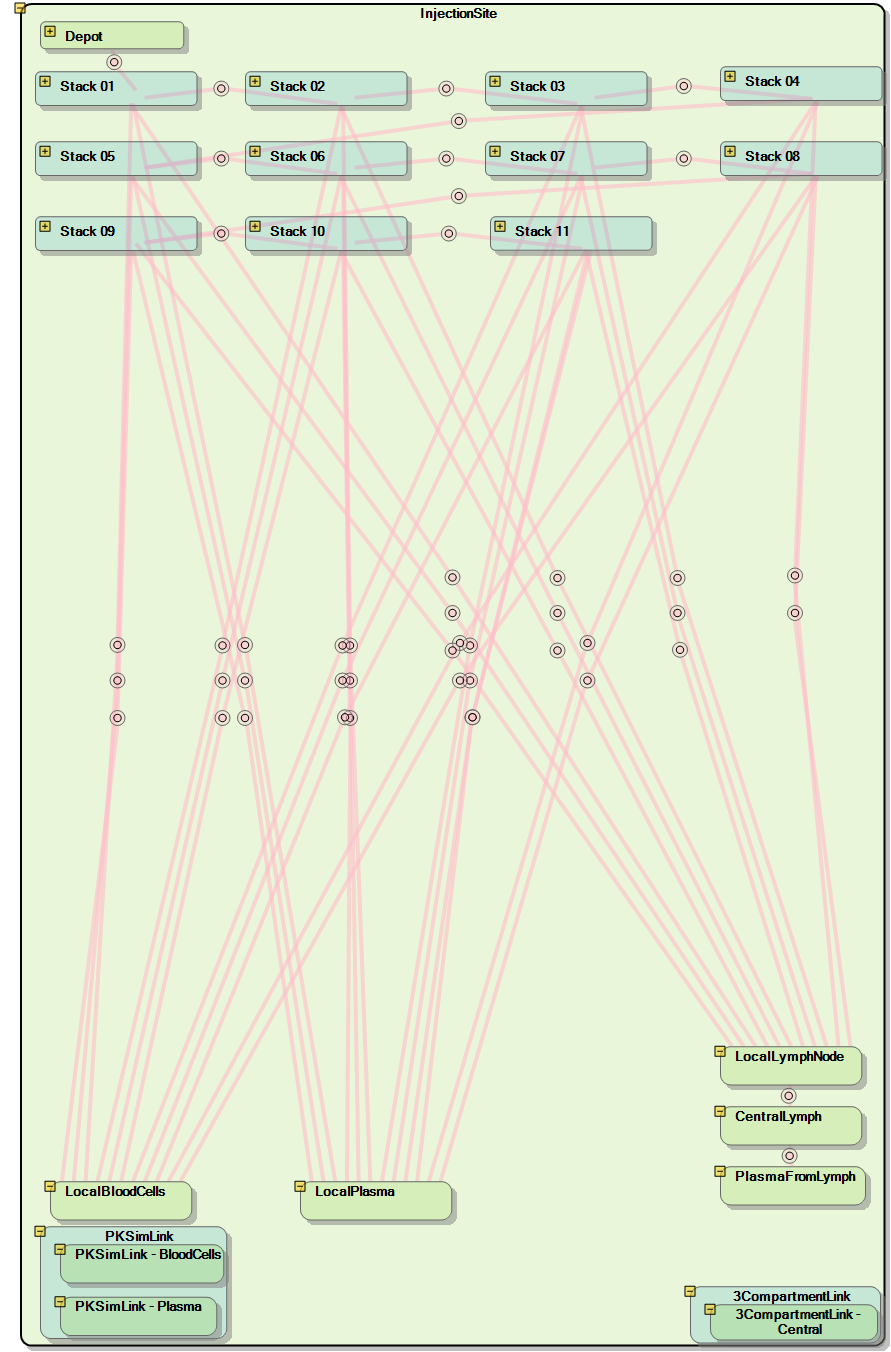 |
| --- | --- |
| b  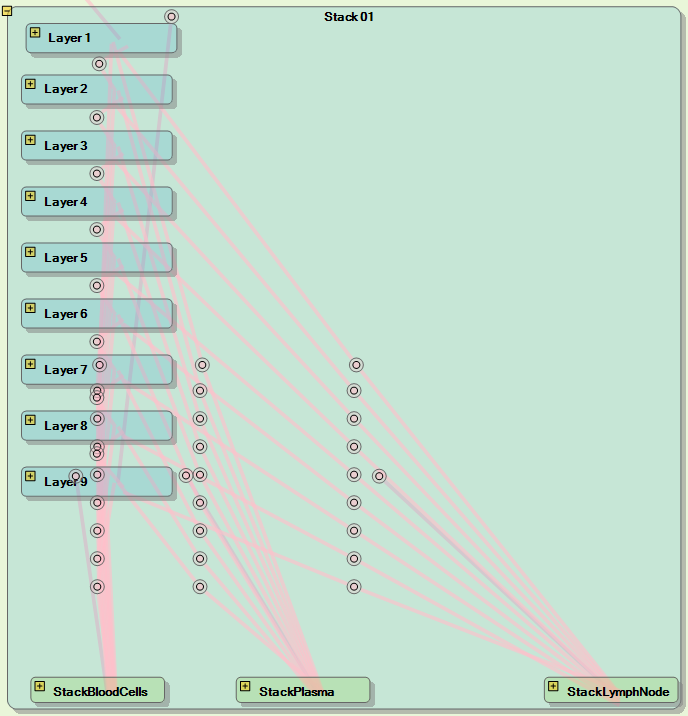 |  |

**Figure S1.1:**

Diagrams of the subcutaneous absorption and disposition model structure, excerpted from the MoBi software. a) Injection site “Layer” structure adopting the generic PK-Sim whole-body physiologically based pharmacokinetic model organ structure. b) Injection site “Stack” structure organizing 9 injection site “Layers”. c) Top level ”InjectionSite” structure encompassing all “Layers” (n=99) organized in “Stacks” (n=11) and the connections to the injection depot “Depot”, lymphatics (“LocalLymphNode” and “CentralLymph”), and compartments facilitating the linking of subcutaneous absorption to systemic disposition models.

1. General methods and software

The subcutaneous model employed the Open Systems Pharmacology (OSP) software (v11.2) and the OSP specific R package *ospsuite*. The model was implemented in the OSP software MoBi, and R is used for efficient model initiation and execution. The subcutaneous model leveraged the default whole-body physiologically based pharmacokinetic (PBPK) implementation and parameterization in PK-Sim. The systemic PBPK model was connected to the subcutaneous model to inform certain compound and physiological parameters in the subcutaneous model.

1. Model Parameterization
   1. Parameters

Table S1.1. Model parameters were informed by “Fat” tissue in the generic PK-Sim physiologically based pharmacokinetic model if not stated otherwise.

| **Section** | **Parameter** | **Description** | **Value or Equation** | **Notes or Source** |
| --- | --- | --- | --- | --- |
| **Injection Site** | $V_{dose}$ | Dose volume | User Input |  |
| **Injection Site** | Injection rate | Injection rate | User Input |  |
| **Injection Site** | ${Is}_{cylinder}$ | Shape of depot | User Input (1/0 - TRUE/FALSE) |  |
| **Injection Site** | N | Number of layers | 99 | Model structure |
| **Injection Site** | $r_{a}$ | Radius of average adipocyte | 37.5 µm | Dauger et al. 2025^1^ |
| **Injection Site** | RCS | Relative concentration in solution in depot | User Input |  |
| **Injection Site** | Protein factor |  | 0.15 | Ellemer et al. 2000 ^2^ |
| **Injection Site** | $V_{filled}$ | Volume filled by injection | $\frac{V_{dose}}{RCS}$ |  |
| **Injection Site** | ER | Elongation ratio | $0.574\cdot\left( \left( \frac{V_{dose}}{f_{v.ER}} \right)\cdot\left( \frac{Injection rate}{f_{q.ER}} \right)^{\frac{1}{3}} \right)^{0.4828}$ | Pepin et al. 2023^3^ |
| **Injection Site** | $f_{q.ER}$ | Factor for Elongation ratio – injection rate | ${10}^{-3}$ mL/min | Pepin et al. 2023^3^ |
| **Injection Site** | $f_{v.ER}$ | Factor for Elongation ratio – volume | $1$ mL | Pepin et al. 2023^3^ |
| **Injection Site** | $L_{Cyl}$ | Cylinder length | $\left( 4\cdot ER^{2}\cdot\frac{V_{filled}}{\pi} \right)^{1/3}$ | if IsCylinder = TRUE |
| **Injection Site** | $L_{thk}$ | Layer thickness | $\left\{ \begin{aligned} \begin{matrix} r_{a} & V_{filled}\leq\min Vol^{1}/3 \end{matrix} \\ \begin{matrix} \frac{3}{N}\cdot\left( \left( \frac{V_{filled}}{\pi\cdot L_{Cyl}} \right)^{\frac{1}{2}}-r_{depot} \right) & IsCylinder=TRUE \end{matrix} \\ \begin{matrix} \frac{3}{N}\cdot\left( \left( \frac{{3\cdot V}_{filled}}{4\cdot\pi} \right)^{\frac{1}{3}}-r_{depot} \right) & IsCylinder=FALSE \end{matrix} \end{aligned} \right.$ |  |
| **Injection Site** | $\min Vol^{1}/3$ | Minimum volume of third of layers | $\left\{ \begin{aligned} \begin{matrix} \pi\cdot{(r_{depot}+\left( \frac{N}{3}\cdot r_{a} \right)}^{2}) \cdot L_{Cyl} & IsCylinder=TRUE \end{matrix} \\ \begin{matrix} \frac{4}{3}\cdot\pi\cdot{(r_{depot}+\left( \frac{N}{3}\cdot r_{a} \right)}^{3}) & IsCylinder=FALSE \end{matrix} \end{aligned} \right.$ |  |
| **Depot** | $r_{depot}$ | Radius of outer boundary | $\left\{ \begin{aligned} \begin{matrix} \sqrt{\frac{V}{\pi\cdot L}} & IsCylinder=TRUE \end{matrix} \\ \begin{matrix} \left( \frac{3\cdot V}{4\cdot\pi} \right)^{\frac{1}{3}} & IsCylinder=FALSE \end{matrix} \end{aligned} \right.$ | V is the depot volume ($V_{depot}$) |
| **Depot** | ${SA}_{depot}$ | Surface area of outer boundary | $\left\{ \begin{aligned} \begin{matrix} 2\cdot r\cdot\pi\cdot(r+L) & IsCylinder=TRUE \end{matrix} \\ \begin{matrix} 4\cdot\pi\cdot r^{2} & IsCylinder=FALSE \end{matrix} \end{aligned} \right.$ | r is the radius of outer boundary ($r_{depot}$) |
| **Depot** | $V_{depot}$ | Volume of the depot | $\frac{{V_{dose}-f_{int} \cdot V}_{filled}}{1-f_{int}}$ |  |
| **Injection Site Layer** | $n$ | Layer number |  | Model structure |
| **Injection Site Layer** | $r_{inner}$ | Radius of inner boundary | $r_{depot}+\left( n-1 \right)\cdot L_{thk}$ |  |
| **Injection Site Layer** | $r_{outer}$ | Radius of outer boundary | $r_{depot}+n\cdot L_{thk}$ |  |
| **Injection Site Layer** | $SA_{out}$ | Surface Area of outer boundary | $\left\{ \begin{aligned} \begin{matrix} 2\cdot\pi\cdot r_{outer}\cdot(r_{outer}+L_{thk}) & IsCylinder=TRUE \end{matrix} \\ \begin{matrix} 4\cdot\pi\cdot r_{outer}^{2} & IsCylinder=FALSE \end{matrix} \end{aligned} \right.$ |  |
| **Injection Site Layer** | $V_{layer}$ | Volume of layer | $V_{outer}-V_{inner}$ |  |
| **Injection Site Layer** | $V_{inner}$ | Volume of inner boundary | $\left\{ \begin{aligned} \begin{matrix} \pi\cdot r_{inner}^{2}\cdot L_{cyl} & IsCylinder=TRUE \end{matrix} \\ \begin{matrix} \frac{4\cdot\pi\cdot r_{inner}^{3}}{3} & IsCylinder=FALSE \end{matrix} \end{aligned} \right.$ |  |
| **Injection Site Layer** | $V_{outer}$ | Volume of outer boundary | $\left\{ \begin{aligned} \begin{matrix} \pi\cdot r_{outer}^{2}\cdot L_{cyl} & IsCylinder=TRUE \end{matrix} \\ \begin{matrix} \frac{4\cdot\pi\cdot r_{outer}^{3}}{3} & IsCylinder=FALSE \end{matrix} \end{aligned} \right.$ |  |
| **Injection Site Layer** | $Q_{b}$ | Blood flow | $V_{layer}\cdot Specific blood flow rate\cdot density$ |  |
| **Injection Site Layer** | $Q_{lymph}$ | Lymph flow | $Lymph flow proportionality factor\cdot Q_{b}\cdot\left( 1-Hct \right)$ | Hct=hematocrit |
| **Injection Site Layer** | $V_{ex}$ | Volume of interstitial space | $V_{layer}\cdot f_{interstitial}$ | Extracellular Fluid |
| **Injection Site Layer** | $V_{cell}$ | Volume of intracellular space | $V_{layer}\cdot f_{intracellular}$ |  |
| **Injection Site Layer** | $V_{p}$ | Volume of plasma | $V_{layer}\cdot f_{vascular}\cdot(1-Hct)$ | Hct=hematocrit |
| **Injection Site Layer** | $V_{bc}$ | Volume of blood cell | $V_{layer}\cdot f_{vascular}\cdot Hct$ | Hct=hematocrit |
| **Local Lymph Node** | $V_{l.node}$ | Volume | $X_{\mathrm{lymph}} = X_{\mathrm{fat}}\cdot X_{h0.lymph}/X_{h0.fat}$  $V_{l.node.h0}=11.35 mL$ | Hu et al. 2020 ^4^ |
| **Local Lymph Node** | $Q_{l.node}$ | Lymph flow | $X_{\mathrm{lymph}} = X_{\mathrm{fat}}\cdot X_{h0.lymph}/X_{h0.fat}$  $Q_{l.cent.h0}=2.78 mL/min$ | Hu et al. 2020 ^4^ |
| **Local Lymph Node** | $k_{eq.l.node}$ | Equilibrium rate constant duct/interstitial | 0.1 L/min | Assumed |
| **Central Lymph** | $V_{l.cent}$ | Volume | $X_{\mathrm{lymph}} = X_{\mathrm{fat}}\cdot X_{h0.lymph}/X_{h0.fat}$  $V_{l.cent.h0}=0.18 L$ | Hu et al. 2020 ^4^ |
| **Central Lymph** | $Q_{l.cent}$ | Lymph flow | $X_{\mathrm{lymph}} = X_{\mathrm{fat}}\cdot X_{h0.lymph}/X_{h0.fat}$  $Q_{l.cent.h0}=2.78 mL/min$ | Hu et al. 2020 ^4^ |

- 1. Volume and flow in local and central lymph nodes

The volume and flow in local and central lymph nodes were adopted from the model parameterization presented by Hu et al (2020) which was based on published data and previous calculations on human lymphatic physiology. ^4–8^ Preclinical parameters were informed by the respective species’ fat physiology, specified by the PK-Sim database, and assuming the same relationship between fat and lymph parameters as in human.

$$X_{\mathrm{lymph}} = X_{\mathrm{fat}} \cdot X_{h0.lymph}/X_{h0.fat}$$

Where X_lymph_ is the parameter in lymph node, X_fat_ is the parameter in fat, and the ratio X_h0.lymph_/X_h0.fat_ represents the relation between the typical parameter values in fat and lymph node in human.

The MOLECULE building block retained the standard implementation as in PK-Sim PBPK model, the following molecular parameters were adjusted locally according to the analysis of respective drug:

- Aqueous diffusion coefficient – depot
- Aqueous diffusion coefficient – layers
- Kd (FcRn) in endosomal space of container
- P_cell_int_factor
- P_int_cell_factor
- Kp_int_ = Partition coefficient (interstitial/plasma)
- Kp_cell_ = Partition coefficient (intracellular/plasma)
- Fraction unbound drug in interstitial fluid via the parameter *Protein factor* according to: $f_{u,isf}=\frac{f_{u}}{f_{u}+Protein factor\left( 1-f_{u} \right)}$
  1. Flows

Indicator: ex / int = extracellular / interstitial), cell = intracellular

- Depot - ExtracellularFluid (first)
  - $\Delta X_{1,ex}=\frac{D{SA}_{depot}}{L_{thk}}\left( c_{depot}-f_{u,isf}c_{1} \right) \Delta t$
- ExtracellularFluid_i - ExtracellularFluid_i+1
  - $\Delta X_{i+1,ex}=\frac{DA_{o,i}f_{u,isf}}{r_{t}}\left( \frac{x_{i,ex}}{V_{i}}-\frac{x_{i+1,ex}}{V_{i+1}} \right)\Delta t$
- ExtracellularFluid - Intracellular
  - As per PK-Sim WB-PBPK model
  - $\Delta X_{i,cell}=SA\left( {Kp}_{int}{\cdot P}_{int-cell}\cdot\frac{x_{i,ex}}{V_{i,ex}}+{Kp}_{cell}{\cdot P}_{cell-int}\cdot\frac{x_{i,cell}}{V_{i,cell}} \right) \Delta t$
- ExtracellularFluid - Plasma
  - According to the 2-pore formalism in PK-Sim.^9^
- ExtracellularFluid - LocalLymphNode
  - $\Delta X_{LocalLymph}=\sum\left( Q_{lymph.i}\cdot\frac{x_{i,ex}}{V_{i,ex}} \right)\Delta t$
- ExtracellularFluid - Endosome
  - According to endosomal recycling and FcRN salvaging in PK-Sim.^10^
- Plasma - Systemic plasma (venous blood)
  - $\Delta X_{plasma}=\sum\left( Q_{b.i}\cdot\left( 1-hematocrit \right)-Q_{lymph.i} \right)\cdot C_{i,plasma}\cdot\Delta t$
- Blood cell - Systemic blood cells (venous blood)
  - $\Delta X_{bloodcell}=\sum Q_{b.i}\cdot hematocrit\cdot C_{i,bloodcell}\cdot\Delta t$
- ExtracellularFluid - MetabolizedDrug
  - $\Delta X_{metabolized}=\sum k_{m}{\cdot f}_{u,isf}\cdot x_{i,ex}\cdot\Delta t$
- LocalLymphNode - CentralLymph
  - $\Delta X_{CentralLymph}=\left( Q_{l.node}\cdot\frac{x_{LocalLymphNode}}{V_{l.node}} \right)\Delta t$
- LocalLymphNode - LocalLymphInterstital
  - $\Delta X_{LocalLymphNode}=k_{eq.l.node}\left( \frac{x_{LocalLymph.ex}}{V_{l.ex}}-\frac{x_{LocalLymphNode}}{V_{l.node}} \right)\Delta t$
- CentralLymph - System (i.e. venous blood)
  - $\Delta X_{System}=\left( k_{l.cent}\cdot X_{CentralLymph} \right)\Delta t$
- Particle dissolution
  - When applicable, dissolution of solid material in the depot was modelled leveraging the built-in particle dissolution model within PK-Sim.^11^
  1. Initial conditions

Table S1.2. Initial conditions (in MoBi called MSVs): The initial amounts of the drug in each layer were set via an R script. The script also determined, based on the input parameters defined by the user, the physical state of the drug, ie, the respective amount solubilized and in solid state.

| **Scenario** | **Condition** | **Calculation** |
| --- | --- | --- |
| **Liquid drug** | $\frac{Initial dose amount}{Dose volume}>Saturation concentration$ | The dissolved drug amount is set by the saturation concentration:  $Liquid Drug (\mu mol) = Dose Volume\cdot Saturation Concentration$ |
|  | $\frac{Initial dose amount}{Dose volume}\leq Saturation concentration$ | The dissolved drug amount is set by the saturation concentration:  $Liquid Drug \left( \mu mol \right)= Initial dose amount$ |
| **Solid drug** |  | $Solid Drug \left( \mu mol \right)= Initial dose amount-liquid drug amount$ |

The initial concentration in the depot compartment was calculated as

$$depot drug \left( umol \right)= liquiddrug\cdot\frac{Vdepot}{Vdose}$$

Initial amounts in interstitial layers: the initial amount was distributed across the surrounding interstitial layers based on whether each layer was saturated by the initial injection volume:

- For saturated layers ($V_{ISF}\left[ i \right]\leq V_{filled}$): The MSV for each layer was calculated proportionally based on its extracellular fluid volume.

$$MSV_{layers}\left[ i \right]= Liquid Drug Amount\cdot\frac{V_{extracellular fluid}\left[ i \right]}{Dose Volume}$$

- For unsaturated layers and subsequent layers: The remaining liquid drug was allocated to the first unsaturated layer

$$MSV_{layer}=Liquid Drug Amount-\sum MSV_{layers}^{Saturated}$$

1. Model Assumptions and implementation
   1. Spatial structure

- It was assumed that the extravascular subcutaneous tissue was made up of a cellular portion (comprised of adipocytes) and an extracellular fluid portion.
- The depot and InterstitialFluid layers were modelled either as concentric spheres or concentric cylinders (with a constant cylinder length). The layer thickness was at least the same as the radius of an adipocyte (arbitrary constraint). It was set so that at most 1/3 of the interstitial fluid layers were filled with the dose volume.
  - Radius and surface area of outer boundary of depot
    - The volume of the depot was calculated based on the fraction of the dose that formed the depot. The radius was then calculated from this volume and used to calculate the surface area of the outer boundary.
  - Layer thickness
    - The volume filled by the injection was calculated as the volume of depot and interstitial fluid layers that were filled initially with dose, assuming that the extracellular fluid compartments and depot were filled by the dose volume. The intracellular fluid compartments were initially empty.
    - The volume filled by the injection was then compared to the volume of a third of the layers, assuming the minimum layer thickness of r_adipocyte_. If the volume filled by the injection was less than the minimum volume of a third, then the layer thickness was set to r_adipocyte_. Otherwise, the layer thickness was set so that the volume of the third of layers was equal to the volume filled by the injection.
  - Volume of layer compartments
    - Note that the volume calculations were self-contained within each interstitial fluid layer, so that they did not depend on other interstitial fluid layers. Instead, they depended only on the layer thickness (which was contained in the InjectionSite compartment) and the layer number.
    - Volumes of sub-compartments were a proportion of the volume of the layer as opposed to the volume of the entire region encompassed by that layer.
- It was assumed that the plasma protein levels (eg, albumin) were a fraction of that which was in the blood. It was currently kept constant based on the parameter “Protein factor”. This value affected the “Fraction unbound in interstitial fluid”, which was set in the Molecule building block.
  1. Flows
- The flows from the depot to the extracellular fluid, between the extracellular fluid layers, and between the extracellular and intracellular compartments were all proportional to the concentration gradient. Note that the volumes of the extracellular fluid compartments corresponded to the extracellular fluid portions of each slice.
- The flow into the systemic circulation was mediated via vascular and lymphatic flow. The extravasation was modelled via the 2-pore formalism and depended on the molecule size and the fraction of unbound drug. Lymphatic drainage affected both free and bound drug.
- Local endosomal clearance was modelled according to the default implementation for large molecules in PK-Sim including FcRn mediated salvaging. The endosomal distribution, uptake and recycling, was assumed to take place via the interstitial space.
- A placeholder process for generic metabolism was included as a flow from the extracellular fluid layers into the MetabolizedDrug compartment. This was described via a first-order rate constant that was set by the user.
- Dissolution was modelled based on the built-in particle dissolution model within PK-Sim. Particle growth from liquid to solid was not expected to occur after administration due to the distribution concentration gradient and that no dynamics to pH was implemented in the current version.
  1. Initial conditions - General
- The user chose the amount of drug being deposited in umol, the dose volume (ie, the volume being injected), the saturated concentration of the compound, and the depot fraction.
- If the solution was unsaturated (amount/volume < saturation concentration), then the particle distribution had to be defined. This required either a normal distribution with mean and sd or a log-normal distribution with geomean and cv. Additionally, the min and max particle radii had to be defined. Optionally, the user could define the number of bins to be any integer between 1 and 19 inclusively. This was not relevant in the context of this analysis and is hence not further described in this document.
- The dose volume and depot fraction were applied directly to the simulation as parameters. Then, the number of layers, volume filled by injection, depot volume, and layer thickness were read from the simulation as the spatial structure updated them according to the spatial structure assumptions.
- First, the solid and liquid drug portions were calculated. Note that solid drug was calculated as the amount of drug that was above the saturation concentration.
- The depot MSV was calculated as the liquid_drug × depot_fraction. Since the liquid within the depot and the extracellular fluid compartments were expected to have the same concentration (with the potential exception of the last extracellular fluid compartment with drug), the amount of drug would be proportional to the volume. Thus, the depot fraction was used to set the depot MSV.
- The volume of the extracellular fluid container was read in from the simulation, and the proportion of liquid drug was set as liquid_drug× (V_ex_ / V_dose_). In other words, the amount of drug was proportional to the amount of volume deposited in that compartment.
  - This was done until the V_ISF_L (ie, the volume enclosed by the outer boundary of the layer) was equal to or less than volume filled by the injection.
  - For the remaining layers (in the first third of layers), the MSV was set to the liquid_drug-sum(MSV_layers). For most layers, this resulted in a value of 0. For the first of these layers, a partial amount may be deposited, resulting in an intermediate concentration for this layer.

1. References

1 Dauger A, Soula H, Audebert C. Adipocyte size distribution: Mathematical model of a tissue property. *Math Biosci*. 2025;384:109433.

2 Ellmerer M, Schaupp L, Brunner GA, et al. Measurement of interstitial albumin in human skeletal muscle and adipose tissue by open-flow microperfusion. *Am J Physiol Endocrinol Metab*. 2000;278:E352-356.

3 Pepin XJH, Grant I, Wood JM. SubQ-Sim: A Subcutaneous Physiologically Based Biopharmaceutics Model. Part 1: The Injection and System Parameters. *Pharm Res*. 2023;40:2195–2214.

4 Hu S, D’Argenio DZ. Predicting monoclonal antibody pharmacokinetics following subcutaneous administration via whole-body physiologically-based modeling. *J Pharmacokinet Pharmacodyn*. 2020;47:385–409.

5 Jr JEM, Bertram CD. Lymphatic System Flows. *Annu Rev Fluid Mech*. 2018;50:459–482.

6 Ying M, Pang BSF. Three-dimensional ultrasound measurement of cervical lymph node volume. *Br J Radiol*. 2009;82:617–625.

7 Varkhede N, Forrest L. Understanding the Monoclonal Antibody Disposition after Subcutaneous Administration using a Minimal Physiologically based Pharmacokinetic Model. *J Pharm Pharm Sci Publ Can Soc Pharm Sci Soc Can Sci Pharm*. 2018;21:130s–148s.

8 Margaris KN, Black RA. Modelling the lymphatic system: challenges and opportunities. *J R Soc Interface*. 2012;9:601–612.

9 Niederalt C, Kuepfer L, Solodenko J, et al. A generic whole body physiologically based pharmacokinetic model for therapeutic proteins in PK-Sim. *J Pharmacokinet Pharmacodyn*. 2018;45:235–257.

10 Niederalt C, Kuepfer L, Solodenko J, et al. A generic whole body physiologically based pharmacokinetic model for therapeutic proteins in PK-Sim. *J Pharmacokinet Pharmacodyn*. 2018;45:235–257.

11 Willmann S, Thelen K, Becker C, Dressman JB, Lippert J. Mechanism-based prediction of particle size-dependent dissolution and absorption: cilostazol pharmacokinetics in dogs. *Eur J Pharm Biopharm Off J Arbeitsgemeinschaft Pharm Verfahrenstechnik EV*. 2010;76:83–94.
