# Supplementary material for: Accelerating Subcutaneous Drug Development: A Mechanistic Absorption Model for the Open Systems Pharmacology Framework
Source: CPT Pharmacometrics Syst Pharmacol. 2026 Jun 25;15(7):e70292. doi: 10.1002/psp4.70292 (PMC13296821; doi:10.1002/psp4.70292)
Supplement: Supplementary file 1 — Data S1: psp470292‐sup‐0001‐DataS1.zip. Supporting Information S1: Subcutaneous Model. Supporting Information S2: Simulation Information and Reference Studies Supporting Information S3: Sensitivity Analyses. [file PSP4-15-e70292-s001.zip › PSP-2026-0008-s02.docx]

Supporting Information S2: Simulation Information and Reference Studies
Accelerating Subcutaneous Drug Development: A Mechanistic Absorption Model for the Open Systems Pharmacology Framework

Table S2.1: Model drug parameters for case example drugs included in study.

| **Drug** | **Class** | **MW^a^** | **pKa (acid)** | **pKa (base)** | **S^b^** | **Species** | **KdFcRn^c^ (µM)** | **fu (%)** | **logP^d^** | **Pc^e^ (10^4^ dm/min)** | **CL_h_^f^**  **(mL/min/kg)** | **Calculation method^g^** | |
| --- | --- | --- | --- | --- | --- | --- | --- | --- | --- | --- | --- | --- | --- |
|  |  |  |  |  |  |  |  |  |  |  |  | **Kp** | **Pc** |
| Buprenorphine | Small | 468 | 10.4 | 8.3 | 16.8 | Human |  | 5 | 3.35 | 6 | 166 | Berezhkovskiy | PK-Sim |
|  |  |  |  |  |  | Dog |  | 5 | 4.25 | 49 | 15 | P&T ^h^ | PK-Sim |
|  |  |  |  |  |  | Rabbit |  | 3 | 4.14 | 38 | 76 | PK-Sim | PK-Sim |
| Fentanyl | Small | 336 |  | 8.8 | 24 | Human |  | 13 | 4.32 | 415 | 11 | P&T ^h^ | PK-Sim |
|  |  |  |  |  |  | Dog |  | 20 | 4.19 | 309 | 10 | Berezhkovskiy | PK-Sim |
| Methotrexate | Small | 454 | 3 | 14 | 81.9 | Human |  | 31 | -0.08 | 0.003 | 123 | R&R ^i^ | PK-sim |
|  |  |  |  |  |  | Monkey |  | 93 | 0.08 | 0.004 | 27 | Berezhkovskiy | PK-Sim |
|  |  |  |  |  |  | Rat |  | 100 | -0.07 | 0.003 | 155 | PK-Sim | PK-Sim |
| Ropivacaine | Small | 274 | 13.6 | 7.8 | 253 | Human |  | 2.3 | 3.33 | 144 | 4.0 | Berezhkovskiy | PK-Sim |
|  |  |  |  |  |  | Rabbit |  | 0.7 | 1.57 | 3 | 17 | R&R ^i^ | PK-Sim |
|  |  |  |  |  |  | Rat |  | 5.5 | 2.16 | 10 | 33 | Schmitt | PK-Sim |
| Lenacapavir | Small | 968 | 6.7 | 1.9 | 0.3 | Dog |  | 0.01 | 6.58 | 502 | 1.0 | P&T ^h^ | PK-Sim dep. ^j^ |
| Adalimumab | Large | 148 |  |  | 1000 | Human | 1.79 | 100 | -5 | 0 | 0 | PK-Sim | PK-Sim |
|  |  |  |  |  |  | Monkey | 0.49 | 100 | -5 | 0 | 0 | PK-Sim | PK-Sim |
|  |  |  |  |  |  | Rat | 2.54 | 100 | -5 | 0 | 0 | PK-Sim | PK-Sim |
|  |  |  |  |  |  | Mouse | 2.03 | 100 | -5 | 0 | 0 | PK-Sim | PK-Sim |
| Rituximab | Large | 143 |  |  | 1000 | Human | 0.77 | 100 | -5 | 0 | 0 | PK-Sim | PK-Sim |
|  |  |  |  |  |  | Monkey | 0.74 | 100 | -5 | 0 | 0 | PK-Sim | PK-Sim |
|  |  |  |  |  |  | Rat | 2.53 | 100 | -5 | 0 | 0 | PK-Sim | PK-Sim |
|  |  |  |  |  |  | Mouse | 1.92 | 100 | -5 | 0 | 0 | PK-Sim | PK-Sim |

^a^ Unit g/mol for Class Small and kDa for Class Large, ^b^ Solubility, Class Small values in µg/ml from ALOGPS ^1^ via DrugBank.com and for Class Large assumed values in mg/ml, all simulations performed applying Solubility in depot = (dose/dose volume)×10, ^c^ Estimated for Class Large, ^d^ Estimated for Class Small and set to -5 for Class Large, ^e^ Calculated for Class Small and set to 0 for Class Large, ^f^ Estimated for Class Small and set to 0 for Class Large,  ^g^ Calculation methods provided in PK-Sim, ^h^ Poulin and Theil, ^i^ Rodgers and Rowland, ^j^ Charge dependent Schmitt normalized to PK-Sim

Table S2.2: Injection parameters and settings for the subcutaneous administration case examples included in the study. Dose and dose volume was informed from respective study report (Table S2.4). In the absence of reliable information, the injection rate and depot geometry was assumed to be 3 ml/min and cylinder, respectively, if not stated otherwise. Precipitation at injection was not allowed.

| **Drug** | **Species** | **Dose (mg)** | **Dose volume (ml)** | **Dispersion (%)** |
| --- | --- | --- | --- | --- |
|  |  |  |  |  |
| Buprenorphine | Human | 0.37/16 | 1^c^/1 ^c^ | 20 ^f^/ 0 ^b^ |
|  | Dog | 0.45 | 0.25 | 60 ^d^ |
|  | Rabbit | 0.16 | 0.52 | 60 ^d^ |
| Fentanyl | Human | 0.2 | 1 ^c^ | 20 ^f^ |
|  | Dog | 0.7 | 10.1 | 60 ^d^ |
| Methotrexate | Human | 15 | 0.3 | 20 ^f^ |
|  | Monkey | 7.45 | 1 ^c^ | 99 ^f^ |
|  | Rat | 1.5 | 1 ^c^ | 52 ^f^ |
| Ropivacaine | Human | 12.5/100 | 2.5/2.5 | 20 ^f^ /20 ^f^ |
|  | Rabbit | 17 | 1 ^c^ | 60 ^d^ |
|  | Rat | 10 | 1 ^c^ | 52 ^f^ |
| Lenacapavir | Dog | 30/60/300/1000 | 0.3/0.6/3/10 | 5/10/67/90 ^b^ |
|  |  |  |  |  |
| Adalimumab | Human | 40 | 0.8 | 20 ^a^ |
|  | Monkey | 16.5 | 3.3 | 99 ^a^ |
|  | Rat | 0.5 | 1 ^c^ | 52 ^a^ |
|  | Mouse | 0.04 | 1 ^c^ | 69 ^a^ |
| Rituximab | Human | 1248 | 9.7 ^e^ | 65 ^a^ |
|  | Monkey | 140 | 1 ^c^ | 99 ^a^ |
|  | Rat | 3.65 | 1 ^c^ | 0 ^a^ |
|  | Mouse | 0.8 | 0.08 | 19 ^a^ |

^a^ Identified in evaluation of dispersion and local endosomal uptake rate
^b^ Manually adopted in formulation investigation
^c^ Assumed
^d^ Average of adalimumab estimates from the included species
^e^ Injection rate 1.9 ml/min
^f^ Informed by identified value for adalimumab in same species

Table S2.3: Local subcutaneous disposition parameters identified for the subcutaneous administration case examples included in the study. Initial (Naïve) parameter values for local partition coefficient (Kp) were informed from Kp.fat as per the model for systemic disposition. Local modulation to cell permeability (P_c.scalar_) and local metabolic rate constant (k_met_) was set to 1 and 0, respectively, if not stated otherwise. The diffusion coefficient in the depot (D_depot_) was specifically adopted for formulation case examples and otherwise set equal to the aqueous diffusion coefficient as calculated by PK-Sim. Local dispersion, scaling of default endosomal uptake rate constant (k_endo.scalar_), and bioavailability was identified for adalimumab and rituximab.

| **Drug** |  | **Naïve** | **1** | **2** | | | **3** | | | **Formulation** |
| --- | --- | --- | --- | --- | --- | --- | --- | --- | --- | --- |
|  | **Species** | **Kp** | **Kp** | **Kp** | **P_c.scalar_** | | **Kp** | **P_c.scalar_** | **k_met_ (min^-1^)** | **D_depot_  (cm^2^/min)** |
| Buprenorphine | Human | 4.0 | 6.1 | 6.1 | 0.0014 | | 11 | 0.0037 | 0.22 | 2.4×10^-7 a^ |
|  | Dog | 12 | 0.10 | 51 | 0.0053 | | 2.9 | 0.012 | 5.5 |  |
|  | Rabbit | 350 | 0.82 | 0.82 | 1.6 | | 0.88 | 0.12 | 0.31 |  |
| Fentanyl | Human | 0.37 | 2.6 | 100^*^ | 0.00024 | | 30 | 0.00016 | 1.9 |  |
|  | Dog | 0.46 | 0.49 | 16 | 0.0001^*^ | | 16 | 0.0001^*^ | 0^*^ |  |
| Methotrexate | Human | 0.1 | 1.2 | 1.1 | 0.36 | | 1.1 | 0.36 | 0^*^ |  |
|  | Monkey | 0.15 | 0.81 | 0.76 | 0.56 | | 0.76 | 0.56 | 0^*^ |  |
|  | Rat | 0.84 | 0.05 | 7.2 | 0.50 | | 2.5 | 0.39 | 0.047 |  |
| Ropivacaine | Human | 4.1 | 0.001^*^ | 0.001^*^ | 1.0 | | 0.17 | 0.82 | 0.82 | 1.0×10^-6 a^ |
|  | Rabbit | 0.0021 | 0.02 | 0.025 | 0.0001^*^ | | 1.6 | 0.18 | 3.1 |  |
|  | Rat | 1.5 | 2.2 | 2.2 | 1.1 | | 1.6 | 5.9 | 0.41 |  |
| Lenacapavir ^b^ | Dog | - | - | - | - | | - | - | - | 1.0×10^-8^ |
|  | | | | | | | | | | |
|  |  | **1** | | **2** | | | | **3** | | |
|  |  | **Dispersion (%)** | | **Dispersion  (%)** | | **k_endo.scalar_** | | **Dispersion (%)** | | **Bioavailability (%)** |
| Adalimumab | Human | 0 | | 20^c^ | | 3.1 | | 34 | | 70 |
|  | Monkey | 99 | | 99^c^ | | 1.6 | | 78 | | 103 |
|  | Rat | 67 | | 52^c^ (78^**^) | | 7.0 (5^*^) | | 64 | | 78 |
|  | Mouse | 91 | | 69^c^ (95^**^) | | 8.0 (5^*^) | | 88 | | 75 |
| Rituximab | Human | 56 | | 65 | | 1.9 | | 71 | | 85 |
|  | Monkey | 99 | | 99 | | 1.2 | | 99 | | 105 |
|  | Rat | 0 | | 0 (0^**^) | | 93 (5^*^) | | 36 | | 12 |
|  | Mouse | 0 | | 19 (0^**^) | | 16 (5^*^) | | 55 | | 53 |

^a^ Adopting Kp, P_c.scalar_ and k_met_ parameter values as identified in setup 3
^b^ Adopting Kp = 5, P_c.scalar_ = 1 and k_met_ = 0 min^-1^
^c^ Carried over to small molecule investigation, see Table S2.2
^*^ 5-fold boundary
^**^ Identified at 5-fold boundary

Table S2.4: Clinical and preclinical reference studies from which data were collated and used either to establish intravenous (IV) physiologically based pharmacokinetic models or as reference data after subcutaneous (SC) dosing.

| **Drug** | **Route** | **Species** | **Reference** |
| --- | --- | --- | --- |
| Buprenorphine | IV | Rabbit | ^2^ |
| Buprenorphine | IV | Dog | ^3^ |
| Buprenorphine | IV | Human | ^4^ |
| Buprenorphine | IV | Dog | ^5^ |
| Buprenorphine | IV | Mice | ^6^ |
| Buprenorphine | IV | Dog | ^7^ |
| Fentanyl | IV | Human | ^8^ |
| Fentanyl | IV | Rat | ^9^ |
| Fentanyl | IV | Human | ^10^ |
| Lenacapavir | IV | Beagle | ^11^ |
| Methotrexate | IV | Human | ^12^ |
| Methotrexate | IV | Rat | ^13^ |
| Methotrexate | IV | Monkey | ^14^ |
| Methotrexate | IV | Monkey | ^15^ |
| Methotrexate | IV | Human | ^16^ |
| Rituximab | IV | Human | ^17^ |
| Rituximab | IV | Rat | ^18^ |
| Rituximab | IV | Monkey | ^19^ |
| Rituximab | IV | Mice | ^20^ |
| Ropivacaine | IV | Human | ^21^ |
| Ropivacaine | IV | Human | ^22^ |
| Ropivacaine | IV | Rabbit | ^23^ |
| Ropivacaine | IV | Rat | ^24^ |
| Ropivacaine | IV | Human | ^25^ |
| Ropivacaine | IV | Human | ^26^ |
| Ropivacaine | IV | Human | ^27^ |
| Adalimumab | IV | Human | ^28^ |
| Adalimumab | IV | Monkey | ^29^ |
| Adalimumab | IV | Rat | ^30^ |
| Adalimumab | IV | Mice | ^31^ |
| Buprenorphine | SC | Rabbit | ^2^ |
| Buprenorphine | SC | Human | ^32^ |
| Buprenorphine | SC | Dog | ^5^ |
| Fentanyl | SC | Human | ^33^ |
| Fentanyl | SC | Dog | ^34^ |
| Methotrexate | SC | Monkey | ^14^ |
| Methotrexate | SC | Human | ^35^ |
| Methotrexate | SC | Rat | ^13^ |
| Methotrexate | SC | Human | ^36^ |
| Ropivacaine | SC | Human | ^37^ |
| Ropivacaine | SC | Rabbit | ^23^ |
| Ropivacaine | SC | Rat | ^38^ |
| Buprenorphine | SC | Human | ^4^ |
| Lenacapavir | SC | Beagle | ^11^ |
| Adalimumab | SC | Rat | ^39^ |
| Adalimumab | SC | Monkey | ^40^ |
| Adalimumab | SC | Human | ^41^ |
| Adalimumab | SC | Mice | ^42^ |
| Rituximab | SC | Rat | ^43^ |
| Rituximab | SC | Mice | ^44^ |
| Rituximab | SC | Monkey | ^19^ |
| Rituximab | SC | Human | ^17^ |

# References

1 Tetko IV, Gasteiger J, Todeschini R, et al. Virtual computational chemistry laboratory--design and description. *J Comput Aided Mol Des*. 2005;19:453–463.

2 Askar R, Fredriksson E, Manell E, et al. Bioavailability of subcutaneous and intramuscular administrated buprenorphine in New Zealand White rabbits. *BMC Vet Res*. 2020;16:436.

3 KuKanich B, Allen P. Comparative pharmacokinetics of intravenous fentanyl and buprenorphine in healthy greyhound dogs. *J Vet Pharmacol Ther*. 2014;37:595–597.

4 Albayaty M, Linden M, Olsson H, Johnsson M, Strandgården K, Tiberg F. Pharmacokinetic Evaluation of Once-Weekly and Once-Monthly Buprenorphine Subcutaneous Injection Depots (CAM2038) Versus Intravenous and Sublingual Buprenorphine in Healthy Volunteers Under Naltrexone Blockade: An Open-Label Phase 1 Study. *Adv Ther*. 2017;34:560–575.

5 Steagall PV, Ruel HLM, Yasuda T, et al. Pharmacokinetics and analgesic effects of intravenous, intramuscular or subcutaneous buprenorphine in dogs undergoing ovariohysterectomy: a randomized, prospective, masked, clinical trial. *BMC Vet Res*. 2020;16:154.

6 Kalliokoski O, Jacobsen KR, Hau J, Abelson KSP. Serum concentrations of buprenorphine after oral and parenteral administration in male mice. *Vet J Lond Engl 1997*. 2011;187:251–254.

7 Barletta M, Ostenkamp SM, Taylor AC, Quandt J, Lascelles BDX, Messenger KM. The pharmacokinetics and analgesic effects of extended-release buprenorphine administered subcutaneously in healthy dogs. *J Vet Pharmacol Ther*. 2018;41:502–512.

8 Lim CBS, Schug SA, Sunderland VB, Paech MJ, Liu Y. A phase I pharmacokinetic and bioavailability study of a sublingual fentanyl wafer in healthy volunteers. *Anesth Analg*. 2012;115:554–559.

9 Ohtsuka H, Fujita K, Kobayashi H. Pharmacokinetics of fentanyl in male and female rats after intravenous administration. *Arzneimittelforschung*. 2007;57:260–263.

10 Parikh N, Goskonda V, Chavan A, Dillaha L. Single-dose pharmacokinetics of fentanyl sublingual spray and oral transmucosal fentanyl citrate in healthy volunteers: a randomized crossover study. *Clin Ther*. 2013;35:236–243.

11 Subramanian R, Tang J, Zheng J, et al. Lenacapavir: A Novel, Potent, and Selective First-in-Class Inhibitor of HIV-1 Capsid Function Exhibits Optimal Pharmacokinetic Properties for a Long-Acting Injectable Antiretroviral Agent. *Mol Pharm*. 2023;20:6213–6225.

12 Stewart CF, Fleming RA, Arkin CR, Evans WE. Coadministration of naproxen and low-dose methotrexate in patients with rheumatoid arthritis. *Clin Pharmacol Ther*. 1990;47:540–546.

13 Kaminskas LM, McLeod VM, Ascher DB, et al. Methotrexate-conjugated PEGylated dendrimers show differential patterns of deposition and activity in tumor-burdened lymph nodes after intravenous and subcutaneous administration in rats. *Mol Pharm*. 2015;12:432–443.

14 Balis FM, Mirro J, Reaman GH, et al. Pharmacokinetics of subcutaneous methotrexate. *J Clin Oncol Off J Am Soc Clin Oncol*. 1988;6:1882–1886.

15 Kuroda T, Namba K, Torimaru T, Kawashima K, Hayashi M. Species differences in oral bioavailability of methotrexate between rats and monkeys. *Biol Pharm Bull*. 2000;23:334–338.

16 Seideman P, Beck O, Eksborg S, Wennberg M. The pharmacokinetics of methotrexate and its 7-hydroxy metabolite in patients with rheumatoid arthritis. *Br J Clin Pharmacol*. 1993;35:409–412.

17 Shpilberg O, Jackisch C. Subcutaneous administration of rituximab (MabThera) and trastuzumab (Herceptin) using hyaluronidase. *Br J Cancer*. 2013;109:1556–1561.

18 Kagan L, Turner MR, Balu-Iyer SV, Mager DE. Subcutaneous absorption of monoclonal antibodies: role of dose, site of injection, and injection volume on rituximab pharmacokinetics in rats. *Pharm Res*. 2012;29:490–499.

19 Mao C-P, Brovarney MR, Dabbagh K, Birnböck HF, Richter WF, Del Nagro CJ. Subcutaneous versus intravenous administration of rituximab: pharmacokinetics, CD20 target coverage and B-cell depletion in cynomolgus monkeys. *PloS One*. 2013;8:e80533.

20 Zhao J, Cao Y, Jusko WJ. Across-Species Scaling of Monoclonal Antibody Pharmacokinetics Using a Minimal PBPK Model. *Pharm Res*. 2015;32:3269–3281.

21 Lee A, Fagan D, Lamont M, Tucker GT, Halldin M, Scott DB. Disposition kinetics of ropivacaine in humans. *Anesth Analg*. 1989;69:736–738.

22 Jokinen MJ, Olkkola KT, Ahonen J, Neuvonen PJ. Effect of ciprofloxacin on the pharmacokinetics of ropivacaine. *Eur J Clin Pharmacol*. 2003;58:653–657.

23 Sawaki K, Okubo M, Shimomiya T, et al. Evaluation of high-performance liquid chromatography and mass spectrometry method for pharmacokinetic study of local anesthetic ropivacaine in plasma. *Biomed Res Tokyo Jpn*. 2009;30:319–324.

24 Mantha VRR, Nair HK, Venkataramanan R, et al. Nanoanesthesia: a novel, intravenous approach to ankle block in the rat by magnet-directed concentration of ropivacaine-associated nanoparticles. *Anesth Analg*. 2014;118:1355–1362.

25 Jokinen MJ, Neuvonen PJ, Lindgren L, et al. Pharmacokinetics of ropivacaine in patients with chronic end-stage liver disease. *Anesthesiology*. 2007;106:43–55.

26 Morton CP, Bloomfield S, Magnusson A, Jozwiak H, McClure JH. Ropivacaine 0.75% for extradural anaesthesia in elective caesarean section: an open clinical and pharmacokinetic study in mother and neonate. *Br J Anaesth*. 1997;79:3–8.

27 Jokinen MJ, Ahonen J, Neuvonen PJ, Olkkola KT. The effect of erythromycin, fluvoxamine, and their combination on the pharmacokinetics of ropivacaine. *Anesth Analg*. 2000;91:1207–1212.

28 Broeder A den, Putte L van de, Rau R, et al. A single dose, placebo controlled study of the fully human anti-tumor necrosis factor-alpha antibody adalimumab (D2E7) in patients with rheumatoid arthritis. *J Rheumatol*. 2002;29:2288–2298.

29 Ng CM, Loyet KM, Iyer S, Fielder PJ, Deng R. Modeling approach to investigate the effect of neonatal Fc receptor binding affinity and anti-therapeutic antibody on the pharmacokinetic of humanized monoclonal anti-tumor necrosis factor-α IgG antibody in cynomolgus monkey. *Eur J Pharm Sci Off J Eur Fed Pharm Sci*. 2014;51:51–58.

30 Park Y, Kim N, Choi J, et al. Qualification and Application of a Liquid Chromatography-Quadrupole Time-of-Flight Mass Spectrometric Method for the Determination of Adalimumab in Rat Plasma. *Pharmaceutics*. 2018;10:61.

31 Piccand M, Bessa J, Schick E, Senn C, Bourquin C, Richter WF. Neonatal Immune Tolerance Induction to Allow Long-Term Studies With an Immunogenic Therapeutic Monoclonal Antibody in Mice. *AAPS J*. 2016;18:354–361.

32 Gralow I, Hornstein WF von, Schleyer E, Hiddemann W. [Pharmacokinetics of buprenorphine in subcutaneous administration]. *Anasthesiologie Intensivmed Notfallmedizin Schmerzther AINS*. 1995;30:412–416.

33 Capper SJ, Loo S, Geue JP, et al. Pharmacokinetics of fentanyl after subcutaneous administration in volunteers. *Eur J Anaesthesiol*. 2010;27:241–246.

34 KuKanich B. Pharmacokinetics of subcutaneous fentanyl in Greyhounds. *Vet J Lond Engl 1997*. 2011;190:e140–e142.

35 Hoekstra M, Haagsma C, Neef C, Proost J, Knuif A, Laar M van de. Bioavailability of higher dose methotrexate comparing oral and subcutaneous administration in patients with rheumatoid arthritis. *J Rheumatol*. 2004;31:645–648.

36 Pachon JA, Kivitz AJ, Heuer K-U, Pichlmeier U. Assessing usability, label comprehension, pen robustness and pharmacokinetics of a self-administered prefilled autoinjector pen of methotrexate in patients with rheumatoid arthritis. *SAGE Open Med*. 2014;2:2050312114564241.

37 Ginosar Y, Haroutounian S, Kagan L, Naveh M, Aharon A, Davidson EM. Proliposomal Ropivacaine Oil: Pharmacokinetic and Pharmacodynamic Data After Subcutaneous Administration in Volunteers. *Anesth Analg*. 2016;122:1673–1680.

38 Shen Y, Ji Y, Xu S, Chen DQ, Tu J. Multivesicular liposome formulations for the sustained delivery of ropivacaine hydrochloride: preparation, characterization, and pharmacokinetics. *Drug Deliv*. 2011;18:361–366.

39 Deng Y, Liu L, Qiang W, Hu L, Wang L, Cheng Z. Methotrexate Reduces the Clearance of Adalimumab by Increasing the Concentration of Neonatal Fc Receptor in Tissues. *Pharm Res*. 2019;36:157.

40 Center for Drug Evaluation and Research. *Biosimilar Multi-disciplinary Evaluation and Review (BMER) BLA 761154 FKB327 (a proposed biosimilar to US-licensed Humira)*. (U.S. Food and Drug Administration, 2020).;at <https://www.accessdata.fda.gov/drugsatfda_docs/nda/2020/761154Orig1s000MultidisciplineR.pdf>

41 Liu Z, Gao Z, Yang W, et al. A randomized, double-blind, single-dose, parallel phase I clinical trial to compare the bioequivalence, immunogenicity, and safety of bevacizumab biosimilar and bevacizumab in healthy Chinese subjects. *Expert Opin Drug Metab Toxicol*. 2022;18:519–527.

42 Oyama S, Ebina K, Etani Y, et al. A novel anti-TNF-α drug ozoralizumab rapidly distributes to inflamed joint tissues in a mouse model of collagen induced arthritis. *Sci Rep*. 2022;12:18102.

43 Kagan L, Mager DE. Mechanisms of subcutaneous absorption of rituximab in rats. *Drug Metab Dispos Biol Fate Chem*. 2013;41:248–255.

44 Kagan L, Zhao J, Mager DE. Interspecies pharmacokinetic modeling of subcutaneous absorption of rituximab in mice and rats. *Pharm Res*. 2014;31:3265–3273.
