# Supplementary material for: Accelerating Subcutaneous Drug Development: A Mechanistic Absorption Model for the Open Systems Pharmacology Framework
Source: CPT Pharmacometrics Syst Pharmacol. 2026 Jun 25;15(7):e70292. doi: 10.1002/psp4.70292 (PMC13296821; doi:10.1002/psp4.70292)
Supplement: Supplementary file 1 — Data S1: psp470292‐sup‐0001‐DataS1.zip. Supporting Information S1: Subcutaneous Model. Supporting Information S2: Simulation Information and Reference Studies Supporting Information S3: Sensitivity Analyses. [file PSP4-15-e70292-s001.zip › PSP-2026-0008-s03.docx]

Supporting Information S3: Sensitivity Analyses
Accelerating Subcutaneous Drug Development: A Mechanistic Absorption Model for the Open Systems Pharmacology Framework


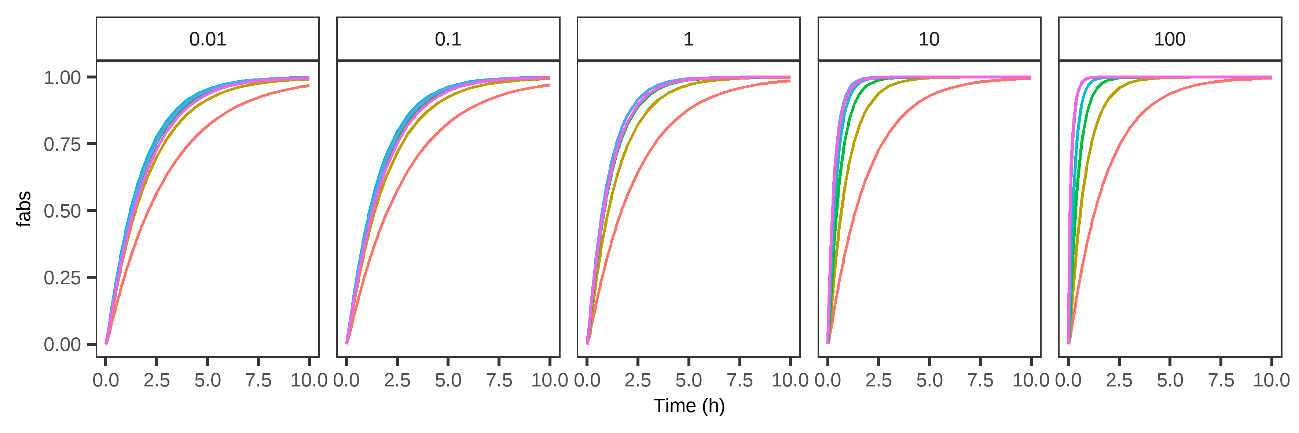


Figure S3.1. Model sensitivity to red blood cell partition coefficient (Kp.rbc) and plasma protein binding for a generic small molecule. Simulated output as total fraction absorbed (fabs) over time for a generic small molecule with variable red blood cell partition coefficient (0.01-100, represented across columns) and fraction unbound in plasma (green = 0.01, orange = 0.025, blue = 0.05, purple = 0.1, red = 0.5, black = 1). Results for fraction unbound equal to 0.5 and 1 are superimposed. For further information see Sensitivity Analysis in Methods.


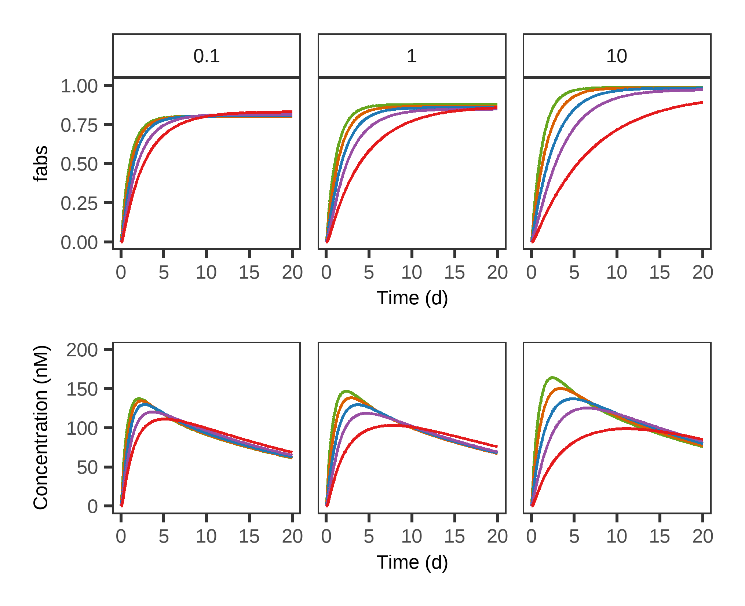


Figure S3.2. Model sensitivity to injection volume and immediate drug dispersion at injection for a generic large molecule. Fraction absorbed (fabs) and plasma concentration over time for a generic large molecule administered with variable injection volume (0.1-10 mL, represented across columns) and immediate drug dispersion at injection (green = 100%, orange = 75%, blue = 50%, purple = 25%, red = 0%). For further information see Sensitivity Analysis in Methods.


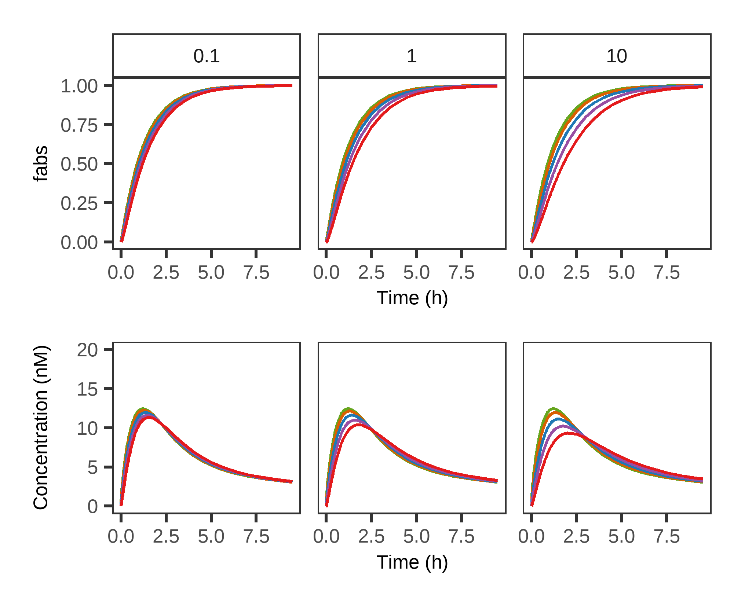


Figure S3.3. Model sensitivity to injection volume and immediate drug dispersion at injection for a generic small molecule. Fraction absorbed (fabs) and plasma concentration over time for a generic large molecule administered with variable injection volume (0.1-10 mL, represented across columns) and immediate drug dispersion at injection (green = 100%, orange = 75%, blue = 50%, purple = 25%, red = 0%). For further information see Sensitivity Analysis in Methods.
